# ROS Produced by NOX2 Controls *In Vitro* Development of Cerebellar Granule Neurons Development

**DOI:** 10.1177/1759091415578712

**Published:** 2015-04-08

**Authors:** Mauricio Olguín-Albuerne, Julio Morán

**Affiliations:** 1 División de Neurociencias, Instituto de Fisiología Celular, Universidad Nacional Autónoma de México, México City, México

**Keywords:** axonal morphogenesis, cerebellar granule neurons, glutathione, NADPH-oxidases, neuronal development, reactive oxygen species

## Abstract

Reactive oxygen species (ROS) act as signaling molecules that regulate nervous system physiology. ROS have been related to neural differentiation, neuritogenesis, and programmed cell death. Nevertheless, little is known about the mechanisms involved in the regulation of ROS during neuronal development. In this study, we evaluated the mechanisms by which ROS are regulated during neuronal development and the implications of these molecules in this process. Primary cultures of cerebellar granule neurons (CGN) were used to address these issues. Our results show that during the first 3 days of CGN development *in vitro* (days *in vitro*; DIV), the levels of ROS increased, reaching a peak at 2 and 3 DIV under depolarizing (25 mM KCl) and nondepolarizing (5 mM KCl) conditions. Subsequently, under depolarizing conditions, the ROS levels markedly decreased, but in nondepolarizing conditions, the ROS levels increased gradually. This correlated with the extent of CGN maturation. Also, antioxidants and NADPH-oxidases (NOX) inhibitors reduced the expression of Tau and MAP2. On the other hand, the levels of glutathione markedly increased at 1 DIV. We inferred that the ROS increase at this time is critical for cell survival because glutathione depletion leads to axonal degeneration and CGN death only at 2 DIV. During the first 3 DIV, NOX2 was upregulated and expressed in filopodia and growth cones, which correlated with the hydrogen peroxide (H_2_O_2_) distribution in the cell. Finally, NOX2 KO CGN showed shorter neurites than wild-type CGN. Taken together, these results suggest that the regulation of ROS is critical during the early stages of CGN development.

## Introduction

The development of the nervous system represents a highly coordinated event that includes different processes such as neural differentiation, neuritogenesis, and programmed cell death. A primary aim of neurobiology is to unravel the molecular mechanisms that control neuronal development that is essential for the correct assembly of neuronal networks (Barnes and Polleux, 2009).

Reactive oxygen species (ROS) have been implicated in several pathological conditions, but they have also emerged as endogenous modulators of numerous physiological functions. ROS are formed from the univalent reduction of molecular oxygen, leading to the production of superoxide anions (${\text{O}}_{2•}^{-}$), considered the primary ROS. Subsequently, the ${\text{O}}_{2•}^{-}$ is converted enzymatically and nonenzymatically into hydrogen peroxide (H_2_O_2_), which is considered the major ROS that regulates cell physiology (Kamata and Hirata, 1999; Droge, 2002; Valko et al., 2007; Pourova et al., 2010).

In the nervous system, ROS have been mainly associated with the cause and progression of different neurologic disorders (Barnham et al., 2004; Sorce and Krause, 2009). Nevertheless, increasing evidence also support a physiological role of ROS in the developing and adult nervous systems. Some major functions of ROS in the central nervous system (CNS) include its involvement in hippocampal long-term potentiation, associative memory (Thiels et al., 2000), as well as its role as neuromodulator of dopamine release in the striatum (Avshalumov et al., 2005, 2008; Sidlo et al., 2008). During nervous system development, different neurogenic regions express high levels of ROS. This expression pattern varies from one brain region to another and is also related to the developmental process at a given time—such as proliferation of neural stem cells (Yoneyama et al., 2010; Dickinson et al., 2011; Le Belle et al., 2011), neurogenesis (Tsatmali et al., 2005, 2006; Le Belle et al., 2011), cell migration (Le Belle et al., 2011), axonal growth (Munnamalai and Suter, 2009; Munnamalai et al., 2014), axonal guidance (Morinaka et al., 2011), and programmed cell death (Valencia and Moran, 2004). The variety of ROS functions in the CNS is wide ranging from cell proliferation to cell death. Interestingly, several studies have shown that the mechanism that governs the physiological action of ROS may be similar to those underlying their pathological actions. Thus, studies pertaining to ROS actions during neuronal development may contribute to the understanding of the development and the disease of the nervous system.

NADPH-oxidases (NOX) family is one of the main ROS forming complexes in neurons. The NOX family comprises seven homologues (NOX 1-5 and DUOX 1-2) that produce superoxide anion and H_2_O_2_ from molecular oxygen. NOX enzymes are widely expressed in most cell types, including neurons and glial cells (Bedard and Krause, 2007; Sorce and Krause, 2009). Although the physiological functions of NOX enzymes in the nervous system are largely unknown, studies have shown that NOX might be involved in some processes such as the early phase of the long-term potentiation in hippocampus (Kishida et al., 2006) and neurogenesis and neuronal maturation.

The members of the NOX family can be activated by several regulators of nervous system development, including growth factors (neurotrophins and fibroblast growth factor [FGF]), cytokines, and the activation of *N*-methyl-d-aspartate receptor (NMDAR), among others (Suzukawa et al., 2000; Kim et al., 2002; Miller et al., 2007; Brennan et al., 2009). On the other hand, previous studies *in vitro* have shown that ROS produced by NOX induce neuronal differentiation of PC12 cells (Suzukawa et al., 2000; Goldsmit et al., 2001; Kamata et al., 2005), SH-SY5Y cells (Nitti et al., 2010), and P19 cells (Kennedy et al., 2010). Furthermore, it has been shown that NOX2 participates in the neurogenesis in the subventricular zone (Le Belle et al., 2011), as well as in the regulation of the actin cytoskeletal dynamics of axonal growth cones in *Aplysia* neurons (Munnamalai et al., 2014). In the developing cerebellum, we recently showed that a transient increase of ROS produced by NOX seems to be involved in the cerebellar foliation and motor function (Coyoy et al., 2013). In view of this, the developing cerebellar cortex constitutes a suitable model for studying the mechanisms by which ROS regulate neuronal development.

The intracellular ROS levels depend not only on the ROS sources but also on the antioxidant systems in the cells. In this regard, the glutathione is a major antioxidant system in the nervous system (Dringen, 2000). Glutathione scavenges a variety of ROS and is an obligated cosubstrate of glutathione peroxidase, which is a major mechanism of defense against H_2_O_2_ (Franco and Cidlowski, 2009; Lubos et al., 2011). In addition, changes in the reduced glutathione (GSH)/oxidized glutathione (GSSG) ratio are considered important determinants in the redox environment and redox signaling in the cells (Schafer and Buettner, 2001; Jones, 2006; Franco and Cidlowski, 2012). Therefore, the actions exerted by ROS during nervous system development may be influenced by the glutathione antioxidant system.

Not much information is available on the glutathione levels during cerebellar development, and the experimental evidence is rather controversial. Rice and Russo-Menna (1998) reported a sudden increase in glutathione from postnatal day 12 that remianed high until the adult. However, other study indicates that the levels of glutathione transiently increase during the first week and then values return to basal levels, remaining relatively low during the subsequent stages of cerebellar development (Nanda et al., 1996).

Although some studies have established a physiological role of ROS during nervous system development, little is known about the interdependence of ROS and NOX enzymes during neuronal development. Furthermore, the mechanisms through which ROS are regulated in the developing neurons remain largely unknown. In this study, we aim to determine the physiological role of ROS in neuronal development of cerebellar granule neurons (CGN) and evaluate the role of NOX and glutathione in the regulation of ROS during this process. To address these issues, we employed cultured CGN that represents a model of study that recapitulates many of the stages of the development of the CGN in the cerebellar cortex *in vivo*. (Powell et al., 1997; Wechsler-Reya and Scott, 1999; Konishi et al., 2004; Ito and Takeichi, 2009). In the cerebellum, postmitotic CGN precursors generate neurites and migrate to the internal granule layer, where they develop dendrites and establish synaptic contacts with mossy fibers (Solecki et al., 2006). The presynaptic inputs at this stage seem to play a key role in the maturation and survival of CGN. The state of depolarization of CGN influences their survival (Gallo et al., 1987), the activation of the enzymes involved in the synthesis of cell-specific neurotransmitters (Caballero-Benitez et al., 2004), and their dendritic pruning and maturation (Shalizi et al., 2006; Ramos et al., 2007).

Here, we determined the changes in the basal levels of ROS in CGN cultures under depolarizing and nondepolarizing conditions throughout CGN development, as well as their effects on CGN maturation. To elucidate the role of glutathione in the developing CGN, we examined the outcome of glutathione depletion on cell viability and axonal integrity. Subsequently, we measured the expression of NOX2 at different stages of CGN development and its localization in these neurons. We also explored the specific production of H_2_O_2_ in growth cones and filopodia. Finally, we evaluated the physiological relevance of ROS, glutathione, and NOX2 during CGN neuritogenesis.

## Materials and Methods

### Materials

Fetal calf serum, penicillin, and streptomycin were from Gibco® (Grand Island, NY, USA). Dihydroethidium, calcein-AM, and MTT were from Molecular Probes® (Eugene, OR, USA). Trizol Reagent, M-MVL Reverse Transcriptase, and oligo (dT)12-18 primer were from Invitrogen™ (Carlsbad, CA, USA). TaqMan® Universal Master Mix II was from Applied Biosystems® (Foster City, CA, USA). Vectashield mounting medium was from VECTOR LABORATORIES (Burlingame, CA, USA). Poly-l-lysine (molecular weight > 300,000), trypsin, DNAse, superoxide dismutase, cytosine-d-arabino-furoanoside, dimethyl sulfoxide (DMSO), 4-(2-aminoethyl) benzenesulfonyl fluoride hydrochloride (AEBSF), dithiobis-2-nitrobenzoic acid (DTNB), glutathione reductase, β-NADPH, glutathione reduced form, glutathione disulfide form, 2-vinylpyridine, buthionine sulphoximine (BSO), EDTA, propidium iodide, and reagents for polyacrylamide gel electrophoresis (PAGE) were obtained from Sigma (St Louis, MO, USA). CDP-Star enhanced chemiluminescence-detecting agent for phosphatase alkaline conjugated antibodies was purchased from Bio-Rad (Hercules, CA, USA). ProSiev QuadColor Protein Marker and Nucleofector VPI-1003 were from Lonza (Basel, Switzerland). Ebselen, and Euk-134 were from Cayman Chemical (Ann Arbor, MI, USA). Apocynin was from Calbiochem (La Jolla, CA, USA). Other chemicals were of the purest grade available from regular commercial sources.

### Antibodies

Antibodies against glyceraldehyde 3-phosphate dehydrogenase (GAPDH) (mab374) were from Millipore (Bedford, MA, USA) and Santa Cruz Biotechnology (Santa Cruz, CA, USA), respectively. Tau (mab4019) and MAP2 (4542) were from Cell Signaling (Danvers, MA, USA). NOX2 (ab31092) was from Abcam (Cambridge, MA, USA). Alexa Fluor® 488 Goat Anti-Rabbit (H+L) and Alexa Fluor® 568 Donkey Anti-Goat IgG (H+L) were from Molecular Probes® (Eugene, OR, USA).

### Cell Culture

All animals used for experimentation described in the present study were treated in accordance with the accepted standards of animal care and with the procedures approved by the local Committee of Research and Ethics of the Instituto de Fisiología Celular, Universidad Nacional Autónoma de México. The protocol used followed the Guidelines for the Care and Use of Mammals in Neuroscience as well as guidelines released by the Mexican Institutes of Health Research and the National Institutes of Health guide for the care and use of laboratory animals. All efforts were made to minimize animal suffering and to reduce the number of animals used.

CGN cultures were prepared from postnatal Day 8 Wistar rats and from postnatal Day 7 NOX2^−/−^ and wild-type mice as previously described (Moran and Patel, 1989). CGN were plated at a density of 265 × 10^3^ cells/cm^2^ in plastic dishes or coverslips coated with poly-l-lysine (5 µg/mL). Culture medium contained basal Eagle's medium supplemented with 10% (v/v) heat-inactivated fetal calf serum, 2 mM glutamine, 25 mM KCl, 50 U/mL penicillin, and 50 mg/mL streptomycin. Cells were maintained under these conditions between 0 and 8 days *in vitro* (DIV). Culture dishes were incubated at 37℃ in a humidified 5% CO2/95% air atmosphere. To prevent the development of nonneuronal cells, cytosine arabinoside (10 µM) was added 24 hr after seeding. At the end of the preparation, CGN cultures contained approximately 95% neurons. The colonies of NOX2 (gp91phox) knockout mice on a C57BL6 background were purchased from The Jackson Laboratory (Bar Harbor, ME, USA) and bred in our Institute.

### Cell Viability

CGN were incubated with calcein (10 µM) and propidium iodide (5 µM) for 15 min at 37℃, and cells were photographed in a fluorescence microscope using filters with the following characteristics: excitation filter wavelength/dichromatic mirror cut-on wavelength/barrier filter wavelengths of 450-490/500/515 and 510-560/565/590 nm for calcein and propidium iodide, respectively. Subsequent analysis involved the determination of immunopositive cells from at least two different images for each condition. Results are expressed as the percentage of viable cells (calcein-positive cells) from the total number of cells (calcein-positive cells + propidium iodide-positive cells) evaluated per field.

### Metabolic Activity

Mitochondrial activity was determined by the conversion of tetrazolium MTT (3-[4,5-dimethylthiazol-2-yl]-2,5 diphenyl tetrazolium bromide) into formazan crystals. CGN were seeded during different days in the same multiwell plate. MTT (0.5 mg/ml) was added to CGN for 15 min at 37℃; the formazan crystals produced from MTT were extracted with 100% DMSO and quantified spectroscopically at 560 nm.

### ROS Detection

CGN were seeded during different days in the same multiwell plate. Cells were washed twice with Locke medium (154 mM NaCl, 25 or 5 mM KCl, 3.6 mM NaHCO_3_, 2.3 mM CaCl_2_, 5.6 mM glucose, and 10 mM HEPES) and then incubated with 3.2 mM of dihydroethidium for 35 min. After that, cells were washed twice with Locke medium, and images were acquired in an epifluorescence microscope using a filter with the following characteristics: excitation filter wavelength/dichromatic mirror cut-on wavelength/barrier filter wavelength of 510-560/565/590 nm. Two images from the same field were acquired, one in phase contrast and the other in fluorescence. Fluorescence measurements of the ethidium cation (E^+^), which is the product of the oxidation of dihydroethidium, were performed tracing a region of interest (ROI) in the soma of 15 cells. These ROI were considered in the fluorescence image, and the mean gray intensity values were averaged. The backgrounds of two different fields were subtracted from the fluorescence values of each measured cell. At least two different images of each condition were evaluated. We measure the fluorescence of 15 cells per photograph, and the average of four wells per experiment was considered as *n* = 1. Results are expressed as absolute values, or data were normalized with respect to 2 DIV.

### Measurement of Glutathione Content

Glutathione was assayed using enzymatic recycling method for a 96 microwell plate (Tietze, 1969; Rahman et al., 2006). CGN were grown on 35 mm culture dishes from 0 to 5 DIV. Cells were washed in PBS and sonicated in 0.1% Triton X-100 and 0.6% sulfosalicylic acid in KPE (0.1 M potassium phosphate buffer with 5 mM EDTA disodium salt, pH 7.5). Next, samples were mixed with equal volumes of DTNB and glutathione reductase, after which 30 s β-NADPH was added. Immediately, kinetic measurements of the absorbance changes at 412 nm were recorded for 15min. Absorbance recordings and the injection of solutions were performed in the microplate reader Synergy HT (BioTek Instruments, USA). To determine GSSG levels, the samples were preincubated with 2.5% vinylpyridine for 60 min and neutralized with triethanolamine prior to kinetic measurement. Glutathione and GSSG standard curves were prepared to calculate the actual concentration in the sample. GSH was calculated from the subtraction of GSSG from the total glutathione.

### Western Blot

CGN were grown on 60 cm culture dishes from 0 to 3 DIV in presence or absence of the antioxidants Ebselen (10 µM) and Euk-134 (20 µM) and the NOX inhibitors AEBSF (50 µM) and apocynin (400 µM) in 24 hr treatments at the indicated times. Cells were washed twice in ice-cold PBS and homogenized in lysis buffer (25 mM Trizma, 50 mM NaCl, 2% Igepal, 0.2% SDS, and complete protease inhibitors, pH 7.4). The protein concentration of cellular homogenates was determined by using the Lowry method. A total of 60 µg of soluble protein per lane was loaded on SDS-PAGE and electrotransferred to polyvinylidene difluoride (PVDF) membranes which were blocked with fat-free milk (5% in Tris-buffered saline (TBS)/Tween 20 (TTBS) buffer [100 mM Trizma, 150 mM NaCl, and 0.1% Tween, pH 7.5]) and incubated overnight at 4℃ with the following specific primary antibodies: 1:3000 mouse anti-GAPDH, 1:500 mouse anti-Tau, and 1:500 rabbit anti-MAP2.

### Measurement of NAPDH-Oxidase Activity in Living Cells

The NAPDH-oxidase activity was determined by extracellular superoxide anion production that was measured by the reduction of cytochrome c. CGN were seeded during different days in the same multiwell plate; cells were washed twice with Locke medium and then were incubated with 100 µl of Locke media containing cytochrome c (250 µM) and β-NADPH (200 µM). Cytochrome c reduction absorbance (550 nm) was recorded at 37℃ in the microplate reader Synergy HT. The amount of superoxide anion released was calculated using an extinction coefficient of 21 mM^−1^ cm^−1^. It was considered 300 µm as the distance that occupies a volume of 100 µl in a well plus the base of the plate.

### Quantitative Real-Time Reverse-Transcription Polymerase Chain Reaction (RT-qPCR)

Total RNA was extracted from CGN grown in depolarizing conditions from 0 to 5 DIV using Trizol Reagent according to the manufacturer's instructions. RNA quality was assessed by denaturing agarose gel electrophoresis and with NanoDro p2000 spectrophotometer (Thermo Scientific, USA). One µg of total RNA from each sample was reverse transcribed into cDNA using M-MVL Reverse Transcriptase with an oligo (dT)12-18 primer. One microgram of cDNA was used to determine the relative gene expression, which was performed in a thermal cycler Rotor-gene 6000 (Corbett Life Science), using TaqMan Universal Master Mix 2X and TaqMan Assay reagent for NOX1 (Rn00586652_m1), NOX2 (Rn00576710_m1), and GAPDH (Rn01775763_g1; Applied Biosystems®, USA). The relative level of amplified mRNA was normalized to the expression of the housekeeping gene GAPDH. The average *C*t value of the endogenous control (GAPDH) for every sample was subtracted from the *C*t value for each target gene, resulting in the Δ*C*_T_ value. Fold change was calculated using the 2^−ΔΔ*C*T^ method where the comparative cycle threshold (ΔΔ*C*_T_) was defined as the difference between Δ*C*_T_ of 1 to 5 DIV minus Δ*C*_T_ of the DIV in which the expression reached its maximum.

### Immunocytochemistry

CGN were plated at a density of 50 × 10^3^ cells/cm^2^ and were grown onto poly-l-lysine-coated glass slides from 0 to 3 DIV. Cells were washed twice in PBS and fixed with 4% paraformaldehyde for 20 min. Subsequently, cells were blocked and permeabilized overnight at 4℃ in blocking solution (PBS containing 0.5% Triton X-100 and 10% normal goat serum) and then were incubated overnight at 4℃ in blocking solution with 1:250 (0.4 µg/ml) rabbit anti-NOX2 and 1:500 mouse anti-Tau antibodies. The primary antibody anti-NOX2 was detected with an Alexa Fluor® 488 goat anti-Rabbit IgG (H+L) secondary antibody (1:1000) incubated for 2 hr at room temperature. The primary antibody anti-Tau was detected with a DyLight 594 goat anti-Mouse IgG (H+L) secondary antibody (1:1000) incubated for 2 hr at room temperature. Coverslips were mounted using Vectashield mounting media with DAPI. Images were captured in the FV10i-LIV confocal microscope (Olympus, USA). Coverslips incubated without primary antibody or with 1:50 (10 µg/ml) normal rabbit IgG showed no staining. The staining for Tau detection did not affect the detection of NOX2 and vice versa.

### Measurements of H_2_O_2_ Localization in Living Cells

CGN were transfected before plating with 5 µg of HyPer-cyto (Evrogen, Rusia), using the Nucleofector program C-13. CGN were visualized in a microscope Axiovert 200 M (Carl Zeiss Microscopy, Germany) with a Lambda DG-4 system (Sutter Instrument, USA) and a PECON system (PECON, Germany) for cell culture and microscopy; an objective Fluar 40x/1.30 Oil M27 was used to visualize the cells. HyPer fluorescence was acquired in two different channels: CH1 (excitation 460–480 nm, beamsplitter 493 nm, emission 505–530) and CH2 (excitation 379–395 nm, beamsplitter 410 nm, emission 465–555) in time-laps imaging. The images are expressed as the ratio of CH1 to CH2 minus their respective backgrounds. A higher index corresponds to higher levels of H_2_O_2_. In order to determine the regions where the levels of H_2_O_2_ were higher, an ROI was traced, and the mean fluorescence intensity was registered during different photograms; the average of these points was normalized with the average of the fluorescence in the soma. The measurements were only considered in the photograms where the ROI was in focus. The measurement of each structure represents *n* = 1. The image analysis was performed in AxioVision 4.8.2 and Image J.

### Axonal Morphology

CGN were labeled with PKH67 in a proportion of 10 × 10^3^ labeled cells and 18 × 10^6^ nonlabeled cells. PKH67 is a dye used for membrane labeling, which is composed by a green fluorescent dye with long aliphatic tales that intercalates into lipid regions of the membranes (Wallace et al., 2008). Cells were treated with BSO (100 µM) at the time of plating and, in some experiments, the cells were treated with Euk-134 (10 µM) 24 hr after plating. Cells were visualized in a fluorescence microscope and photographed. The morphology of the axons was classified into three different categories that were as follows: unaltered, with multiple spheroids, and collapsed. The proportion of neurons categorized into these three types of morphologies was determined per experiment and averaged in independent experiments.

### Axonal Growth

CGN were labeled before plated with 3 µM PKH67 at room temperature for 5 min. An equal volume of 1% BSA in PBS was then added, and the cells were further incubated for 1 min to stop the staining reaction. The labeled cells were then centrifuged at 400 g for 8 min at room temperature and resuspended in culture medium in a proportion of 10 × 10^3^ labeled cells and 18 × 10^6^ nonlabeled cells. The neurites were measured manually, and only those neurites longer than the cell diameter and that did not show contacts with other neurites were considered; in the case of bipolar CGN, only the longest neurite was considered. The measurement of each neurite represents *n* = 1. The measurements were made in Image J with the plugin Neuron J.

### Statistical Analysis

Statistical analysis was done by using SigmaPlot 12.1 software. Data are expressed as means ± *SEM*, unless otherwise indicated. Pairwise comparison within multiple groups was done by analysis of variance (ANOVA) followed by the Holm–Sidak post hoc test; in some cases, ANOVA nonparametric test was performed. The statistical significance in the comparisons between two groups was determined by the Student's *t* test and by the Mann–Whitney U test. For the time course results, a paired *t* test was used; *p* values less than 0.05 were considered statistically significant.

## Results

### The Levels of ROS Are Differentially Regulated During CGN Development

**Figure 1.**
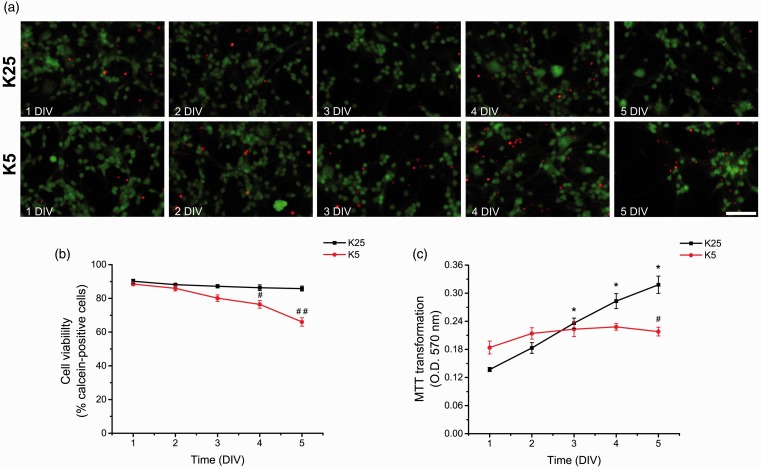

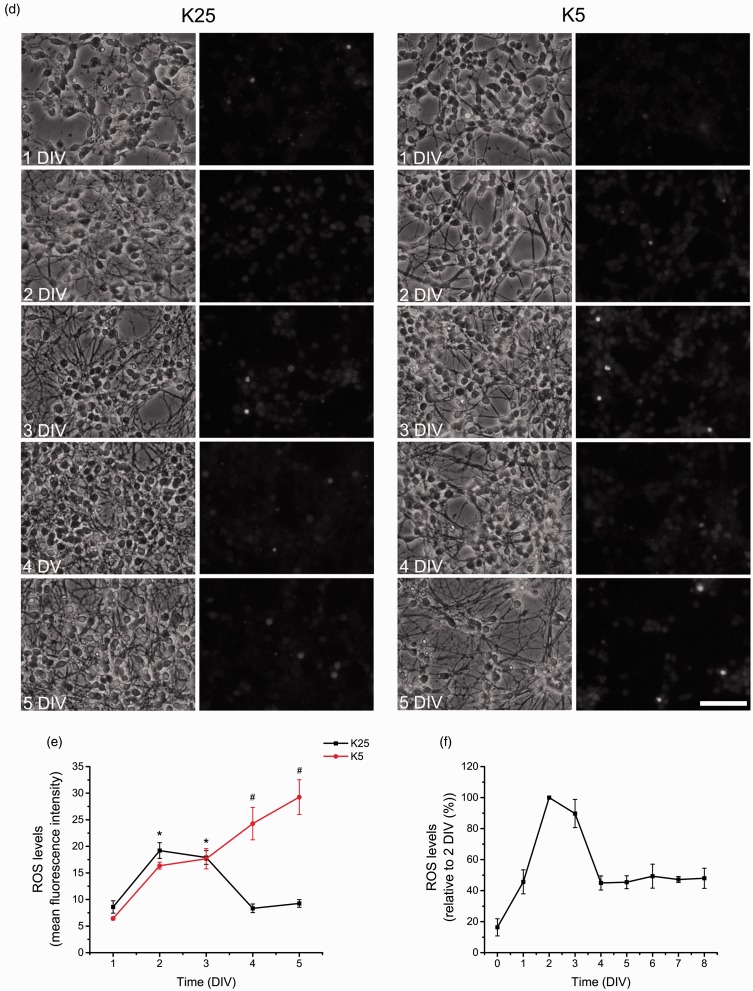
ROS are differentially produced during CGN development. (a) Representative micrographs of CGN grown in depolarizing conditions (K25) and nondepolarizing conditions (K5) from 1 to 5 DIV. Calcein-positive cells are marked in green, and propidium iodide-positive cells are marked in red (scale bar, 100 µm). (b) Cell viability is expressed as the percentage of calcein-positive cells from the total number of cells, which was estimated as the sum of calcein-positive cells plus propidium iodide-positive cells. #, ## are significantly different from K25 at 4 and 5 DIV (*p* < .01, *p* < .001, ANOVA, *n* = 6). Data are mean ± *SEM*. (c) Metabolic activity was determined by MTT transformation of CGN grown in K25 or K5 from 1 to 5 DIV. *is significantly different from K25 at 1 DIV (*p* < .001, ANOVA, *n* = 6). # is significantly different from K25 at 5 DIV (*p* < .001, ANOVA, *n* = 6). Data are mean ± *SEM*. (d) Phase contrast and fluorescence micrographs of CGN grown in K25 or K5 during 1 to 5 DIV (scale bar, 100 µm). (E) ROS levels of CGN grown in K5 or K25 from 1 to 5 DIV. ROS levels are expressed as mean values of the mean fluorescence intensity of ethidium cation, which is the product of the dihydroethidium oxidation. * is significantly different from K25 at 1 DIV (*p* < .05, ANOVA, *n* = 4). # is significantly different from K25 at 4 and 5 DIV (#*p* < .001, ANOVA, *n* = 4). Data are mean ± *SEM*. (f) ROS levels of CGN grown in K25 from 0 to 8 DIV. ROS levels produced from 1 to 8 DIV were higher compared to 0 DIV (*p* < .001, ANOVA, *n* = 3–9). ROS levels produced at 2 and 3 DIV were higher compared to 0, 1, 4–8 DIV (*p* < .001, ANOVA, *n* = 3–9). Data are normalized with respect to 2 DIV and are presented as mean ± *SD*. DIV = days *in vitro*; ROS = reactive oxygen species; CGN = cerebellar granule neurons; ANOVA = analysis of variance.

In order to determine the basal levels of ROS throughout CGN development, we cultured CGN in depolarizing (25 mM KCl) and nondepolarizing (5 mM KCl) concentrations of potassium chloride. This enabled us to compare two conditions of neuronal development *in vitro* where neurons under depolarizing conditions develop normally, while those in nondepolarizing conditions mature only partially and died by 8 DIV in a ROS-dependent manner (Schulz et al., 1996). Under these conditions, we measured the cell viability and the metabolic activity of CGN during the first 5 DIV in order to determine the time at which cell survival is compromised during CGN development. In agreement with Gallo et al. (1987) and others, CGN survival, measured as calcein-propidium iodide incorporation, was dependent on the state of depolarization after 3 DIV. Figure 1 shows that when CGN were cultured in nondepolarizing conditions, the cell viability diminished from 4 DIV, while in depolarizing conditions, the cell viability remained unaltered (Figure 1(a) and (b)). On the other hand, the metabolic activity, measured as MTT transformation, was reduced by 30% at 5 DIV in nondepolarizing conditions (Figure 1(c)). In contrast, the metabolic activity of CGN grown in depolarizing conditions showed a sustained increase during the first 5 DIV (Figure 1(c)).

We also evaluated the basal levels of ROS by the oxidation of dihydroethidium in depolarizing and nondepolarizing conditions during the first 5 DIV (Figure 1(d)). When CGN were cultured in depolarizing conditions, the levels of ROS increased steadily over time from 0 to 1 DIV (160%) and from 1 to 2 DIV (110%). The maximum levels reached at 2 DIV were sustained until 3 DIV (Figure 1(e) and (f)). Subsequently, the levels of ROS decreased to similar values to those found at 1 DIV (Figure 1(e)). Then, the levels of ROS remained low until 8 DIV (Figure 1(f)). On the other hand, when CGN were cultured in nondepolarizing conditions, the levels of ROS were similar to those observed in depolarizing conditions from 1 to 3 DIV (Figure 1(e)); however, at 4 and 5 DIV, the levels of ROS increased gradually (Figure 1(e)). Thus, under these conditions, ROS levels show two distinct phases: the first phase, from 0 to 3 DIV, which is independent of the state of depolarization, and a second phase, after 3 DIV, which is dependent on the state of depolarization.

### Increased Glutathione Levels Are Required for CGN Survival During Early Development

**Figure 2.**
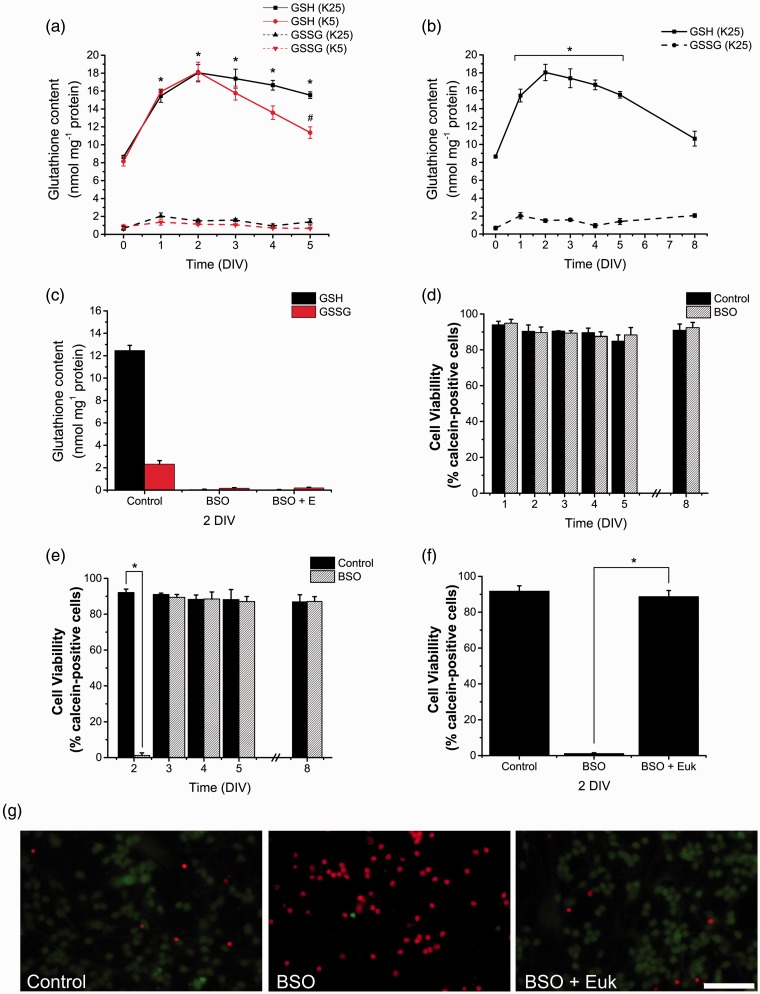
Glutathione is differentially produced and is necessary for CGN survival. (a, b) Reduced glutathione (GSH) and oxidized glutathione (GSSG) content were determined in CGN grown in K25 and K5 from 0 to 8 DIV by a modification of the Tietze recycling assay as detailed in Methods. (a) * is significantly different from K25 at 0 DIV (*p* < .001, ANOVA, *n* = 4). # is significantly different from K25 at 5 DIV (*p* < .001, ANOVA, *n* = 4). Data are mean ± *SEM*. (b) * is significantly different from K25 at 0 DIV (*p* < .001, ANOVA, *n* = 4). Data are mean ± *SEM*. (c) GSH and GSSG were determined in CGN grown in K25 at 2 DIV and treated with BSO (100 µM) for 48 hr and Euk-134 (10 µM) for 24 hr. BSO treatments reduced the levels of GSH and GSSG (*p* < .05, ANOVA nonparametric test, *n* = 5). Data are mean ± *SEM*. (d to f) Cell viability was determined by calcein and propidium iodide. Data are expressed as the percentage of calcein-positive cells from the total number of cells, which was estimated as the sum of calcein-positive cells plus propidium iodide-positive cells. (d) Cell viability was determined in CGN grown in K25 and treated with BSO (100 µM) for 24 hr at 1, 2, 3, 4, 5, and 8 DIV (no statistical differences were found, ANOVA, *n* = 4). (e) Cell viability was determined in CGN grown in K25 and treated with BSO (100 µM) for 48 hr at 2, 3, 4, 5, and 8 DIV. * is significantly different from Control at 2 DIV (*p < .001, ANOVA, *n* = 4). (f) Cell viability was determined in CGN grown in K25 at 2 DIV and treated with BSO (100 µM) for 48 hr and Euk-134 (10 µM) for 24 hr. * is significantly different from BSO at 2 DIV (*p* < .001, ANOVA, *n* = 4). Data are mean ± *SEM*. (g) Representative micrographs of CGN grown in K25 and treated with BSO (100 µM) for 48 hr and Euk-134 (10 µM) for 24 hr. Calcein-positive cells are marked in green and propidium iodide-positive cells are marked in red (scale bar, 100 µm). ANOVA = analysis of variance; BSO = buthionine sulphoximine; CGN = cerebellar granule neurons; DIV = days *in vitro*.

The levels of ROS in the cell are determined by the equilibrium between the production of ROS and the levels and activity of the antioxidants (Halliwell, 2009). Among the numerous antioxidants, we evaluated the glutathione system that is one of the most relevant antioxidant systems in neurons. Moreover, it has been reported glutathione content changes during the cerebellar cortex development (Nanda et al., 1996; Rice and Russo-Menna, 1998). Thereby, in order to evaluate a possible relationship between glutathione and ROS levels throughout CGN development, we estimated the levels of GSH and GSSG throughout CGN development in depolarizing and nondepolarizing conditions. Figure 2(a) shows that when CGN were grown in depolarizing conditions, the GSH content increased during the first 2 DIV. It is important to note that the most significant increase was recorded between 0 and 1 DIV (∼90%) that was followed by a slight increase of 10% between 1 and 2 DIV, which persisted until 5 DIV. At 8 DIV, the levels of GSH significantly decreased to values similar to those observed at 0 DIV (Figure 2(b)). When cells were grown in nondepolarizing conditions, the increase in the levels of GSH was similar to that in depolarizing conditions from 0 to 2 DIV; however, after 3 DIV, the level of GSH decreased significantly in CGN under nondepolarizing conditions at 5 DIV (Figure 2(a)). Interestingly, the level of GSSG did not change throughout the different stages of CGN development, and no significant difference was observed between depolarized and nondepolarized cells (Figure 2(a).

Considering that the levels of glutathione increase in correspondence with ROS, we hypothesized that glutathione might be involved in the actions of ROS during the first 3 DIV. This is further supported by previous studies showing that high levels of ROS induce cell death in these cells. It is possible that the observed decrease in glutathione content in CGN maintained in nondepolarizing conditions during 3 DIV could either be a cause or the result of the cell death observed at this time. In this regard, it has been shown that glutathione depletion triggers cell death and that glutathione depletion occurs during the cell death process (Franco and Cidlowski, 2009, 2012).

**Figure 3.**
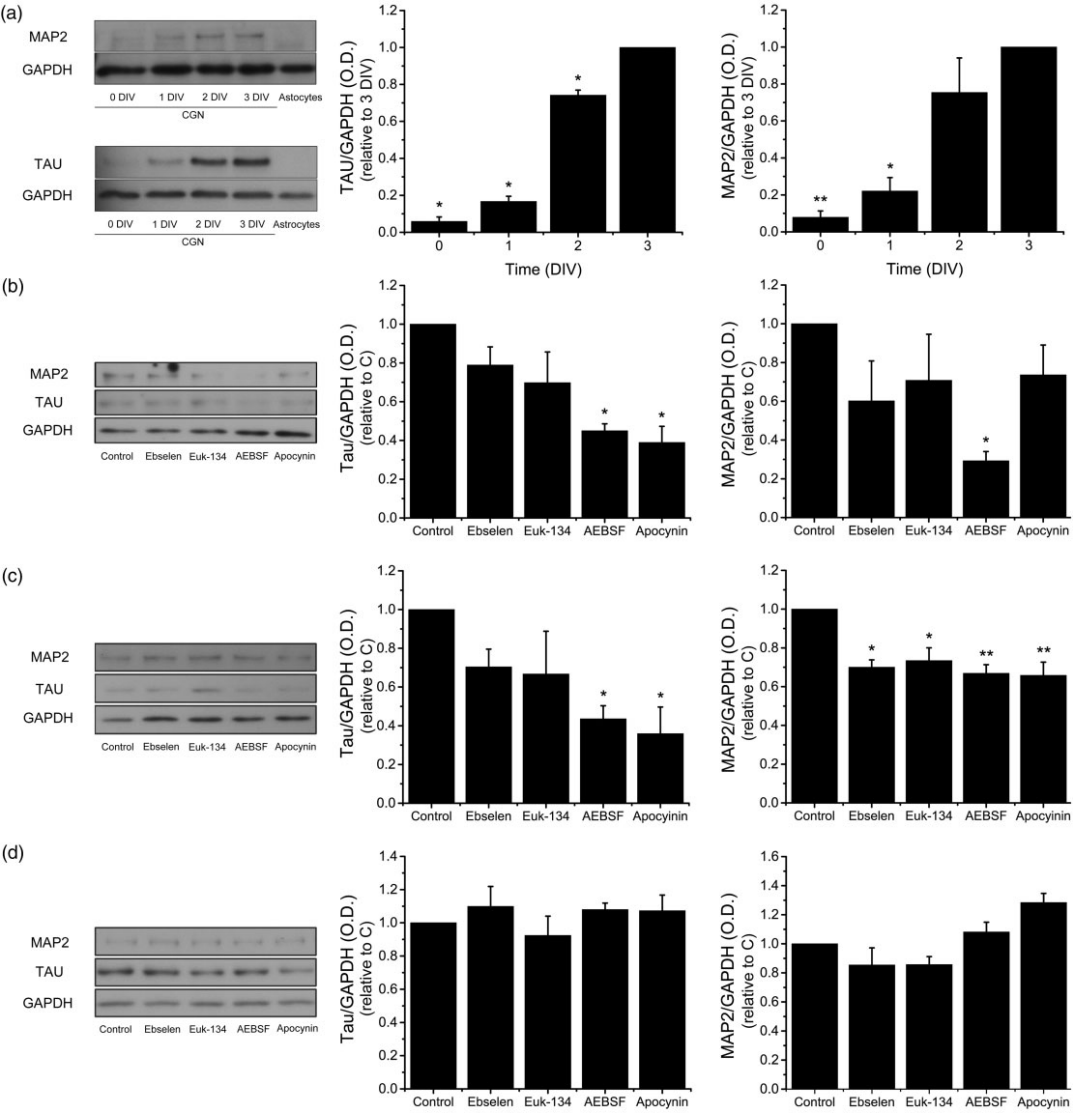
NOX-produced ROS promote CGN maturation. The levels of Tau and MAP2 were determined by Western blot in homogenates of CGN grown in K25 from 0 to 3 DIV and cultured cerebellar astrocytes. (a) Representative blots of Tau (∼70 kDa) and MAP2 (∼280 kDa) with their respective densitometric analysis of Tau (*p < .001, ANOVA, *n* = 3) and MAP2 (**p < .005, *p < .001, ANOVA, *n* = 4). Data were normalized with respect to 3 DIV and are mean ± *SEM*. (b to d) Representative blots of Tau and MAP2 with their respective densitometric analysis of CGN treated with the antioxidants Ebselen (10 µM) or Euk-134 (20 µM) or the NOX inhibitors AEBSF (50 µM) or Apocynin (400 µM) for 24 hr. (b) CGN at 1 DIV, Tau (*p < .001, ANOVA, *n* = 5), MAP2 (*p < .01, ANOVA, *n* = 4). (c) CGN at 2 DIV, Tau (*p < .05, ANOVA, *n* = 4), MAP2 (*p < .01, ** *p* < .001, ANOVA, *n* = 4). (d) CGN at 3 DIV, Tau, and MAP2, no statistical differences were found (ANOVA, *n* = 5). Densitometric values are the ratio of Tau/GADPH or MAP2/GAPDH and are normalized with respect to control. Data are mean ± *SEM*. NOX = NADPH-oxidase; ROS = reactive oxygen species; CGN = cerebellar granule neurons; ANOVA = analysis of variance; DIV = days *in vitro*.

Based on these evidences, we evaluated the role of glutathione in CGN survival by reducing the glutathione content in CGN cultures treated during the last 24 hr or 48 hr of culture with BSO, an inhibitor of γ-glutamylcysteine synthetase, which is the first and rate-limiting enzyme for the biosynthesis of glutathione. Figure 2(c) and Supplementary Figure 1 show that BSO treatment for 48 hr, markedly decreased GSH content at 2, 3, 5, and 8 DIV. Similarly, GSSG content were also significantly reduced by BSO at all ages measured. When cell viability was evaluated, we found that BSO did not affect CGN survival at any time when cultures were treated during the last 24 hr (Figure 2(d); however, when cultures were treated for 48 hr, we observed a significant complete reduction of cell viability in CGN cultures at 2 DIV (Figure 2(e). The observed effect of BSO was completely prevented by the antioxidant Euk-134 (Figure 2(f) and (g)), suggesting that cell death induced by BSO at 2 DIV is due to an oxidative stress induced by glutathione reduction. Interestingly, under these conditions, cell viability was not affected in cultures of 3 to 8 DIV. These results indicate that CGN vulnerability to cell death related to oxidative stress is present only at 2 DIV, when CGN are in the process of maturation. The fact that the levels of glutathione were also completely diminished in CGN treated with BSO plus Euk-134 at 2 DIV (Figure 3(c)), a condition that does not affect cell viability, indicates that glutathione estimation was not affected by the antioxidant nor by the low protein content induced by the cell death process. Thus, there seems to be a narrow developmental time window where ROS seem to play a critical role not only for neuronal survival but also for neuronal maturation. In addition, from 3 to 8 DIV, ROS are no longer required for cell maturation, but the lack of trophic influences (nondepolarizing conditions) could induce some mechanisms leading to an increase in ROS levels and then to cell death.

### ROS Promote CGN Development

Previous studies suggest that ROS regulate some events of the nervous system development (Tsatmali et al., 2006; Le Belle et al., 2011; Celotto et al., 2012; Coyoy et al., 2013). In order to evaluate the relevance of ROS during CGN development, we tested the effect of two antioxidants, the mimetic of glutathione peroxidase Ebselen (Parnham and Kindt, 1984) and the mimetic of superoxide dismutase and catalase Euk-134 (Baker et al., 1998), as well as two general inhibitors of NOX1-2, AEBSF and apocynin (Diatchuk et al., 1997; Cheret et al., 2008; Hernandez-Enriquez et al., 2011; Dvoriantchikova et al., 2012; Lu et al., 2012) on the expression of Tau and MAP2 during CGN development. The expressions of these proteins are indicators of neuronal maturation (Dehmelt and Halpain, 2004). First, we determined the relative levels of Tau and MAP2 from 0 to 3 DIV, and, as expected, we found a correlation between the levels of these two proteins and the CGN development (Figure 3(a)). In both cases, the highest increase was between 1 and 2 DIV. When CGN were treated with Ebselen and Euk-134 at 0 DIV during 24 hr, no effect on the levels of Tau and MAP2 were observed; however, AEBSF and apocynin significantly diminished the levels of Tau by 50%, while AEBSF, but not apocynin treatment, significantly diminished the levels of MAP2 by 70% (Figure 3(b)). When CGN were treated during 24 hr at 1 DIV, the antioxidants had no effect on the levels of Tau, but diminished MAP2 levels by 30%. In contrast, both AEBSF and apocynin significantly reduced the expression of Tau and MAP2 by 60% and 30%, respectively (Figure 3(c)). Finally, when CGN were treated from 2 to 3 DIV, none of the treatments had any effect in the levels of Tau and MAP2 (Figure 3(d)).

### NADPH Oxidase Activity and the Expression of NOX Homologues Change During CGN Development

**Figure 4.**
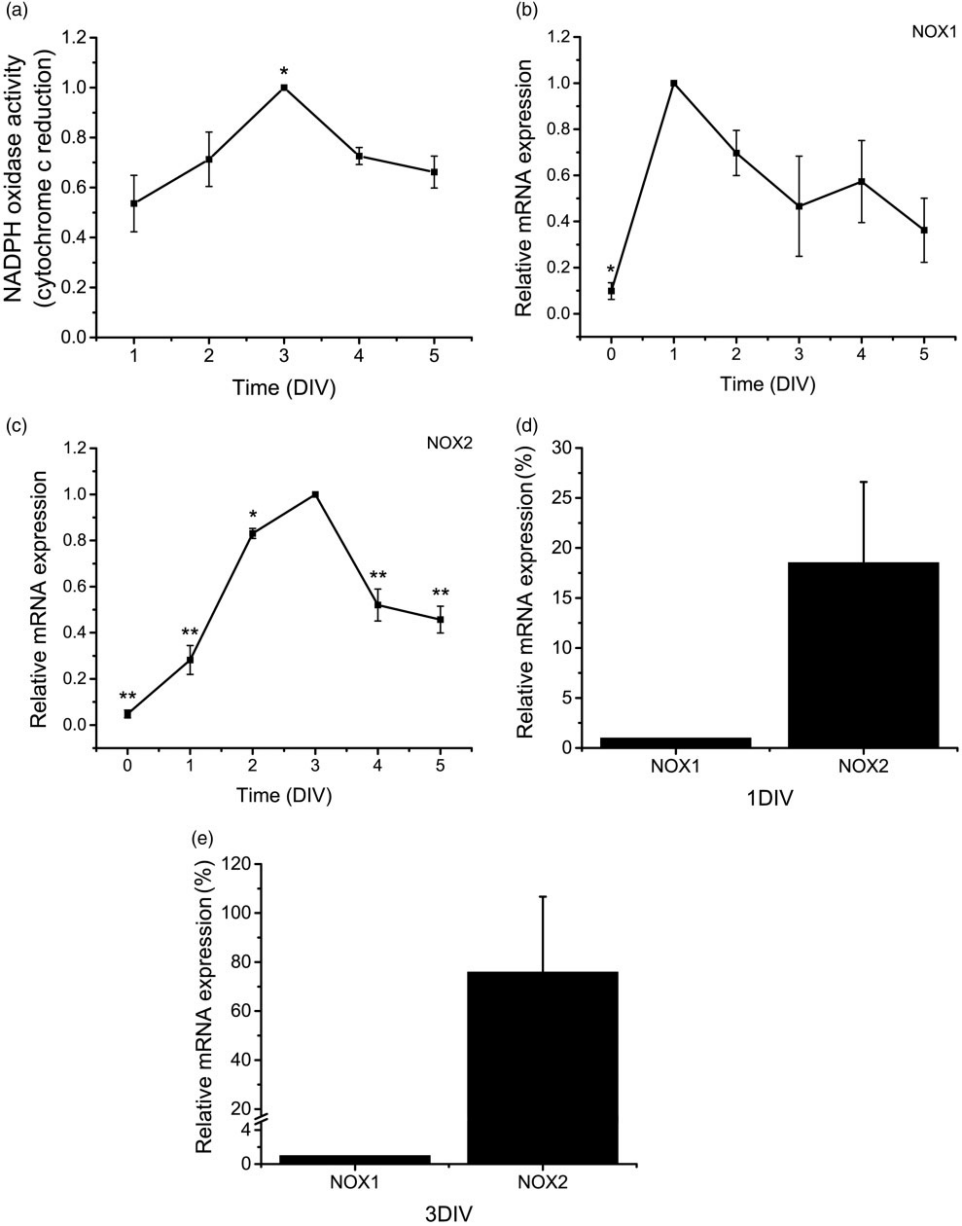
NOX1 and NOX2 are differentially expressed during CGN development. (a) NOX activity was determined in CGN from 1 to 5 DIV by cytochrome c reduction as detailed in Methods. * is significantly different from 1 DIV (*p* < .01, ANOVA, *n* = 5). Data were calculated as nmol min^−1^ per mg protein and were normalized with respect to 3 DIV and are presented as mean ± *SEM*. (b to e) Relative mRNA levels of NOX1 and NOX2 were determined in CGN from 0 to 5 DIV by the 2^−ΔΔCt^ method of relative quantification as detailed in Methods. Data were normalized with respect to the time in which the expression reaches its maximum level. (b) * is significantly different from 1 DIV (*p* < .01, ANOVA, *n* = 3). Data are mean ± *SEM*. (c) NOX2 mRNA levels at 0, 1, 2, 4, and 5 DIV were significantly different with respect to 3 DIV (*p < .05, ** *p* < .001, ANOVA, *n* = 3). Data are mean ± *SEM*. (d) NOX2 mRNA levels at 1 DIV are significantly different from NOX1 mRNA levels at 1 DIV (*p* < .05, Mann–Whitney U Test, *n* = 4). Data are mean ± *SEM*. (e) NOX2 mRNA levels at 3 DIV are significantly different from NOX1 mRNA levels at 3 DIV (*p* < .05, Mann–Whitney U Test, *n* = 4). Data are mean ± *SEM*. NOX = NADPH-oxidase; CGN = cerebellar granule neurons; DIV = days *in vitro*; ANOVA = analysis of variance.

It has been shown previously that NOX enzymes are major source of ROS in several cell types (Bedard and Krause, 2007; Sorce and Krause, 2009). Because NOX inhibitors interfere with CGN maturation, we hypothesized that members of the NOX family could be mediating ROS production during CGN development. In order to determine the relevance of ROS producing enzymes in developing CGN, we measured the NOX activity in cultured CGN by determining the extracellular reduction of cytochrome c by superoxide anion produced by NOX (Figure 4(a)). In CGN grown under depolarizing conditions, the NOX activity gradually increased between 1 to 3 DIV (∼50%) and then the activity decreased to levels close to those found at 2 DIV, which is similar to the ROS production observed from 1 to 5 DIV (Figure 1(e)).

To evaluate the importance of some NOX homologues in the CGN development, we determined the expression of NOX1 and NOX2 in CGN cultured in depolarizing conditions from 0 to 5 DIV by using quantitative real-time RT-PCR (Figure 4(b) to (e)). Under these conditions, we found that NOX1 and NOX2 showed the lowest expression at 0 DIV. NOX1 reached the highest expression at 1 DIV and afterwards it gradually decreased (Figure 4(b)). NOX2 presented a marked increase from 1 to 2 DIV and a minor increment between 2 and 3 DIV. Afterwards, the levels of NOX2 diminished abruptly from 3 to 4 DIV (Figure 4(c)). When the relative mRNA expression of NOX1 and NOX2 were compared, we observed a higher abundance of NOX2 than NOX1 at both 1 and 3 DIV (Figure 4(d) and (e)).

### NOX2 and H_2_O_2_ Localization in Developing CGN

**Figure 5.**
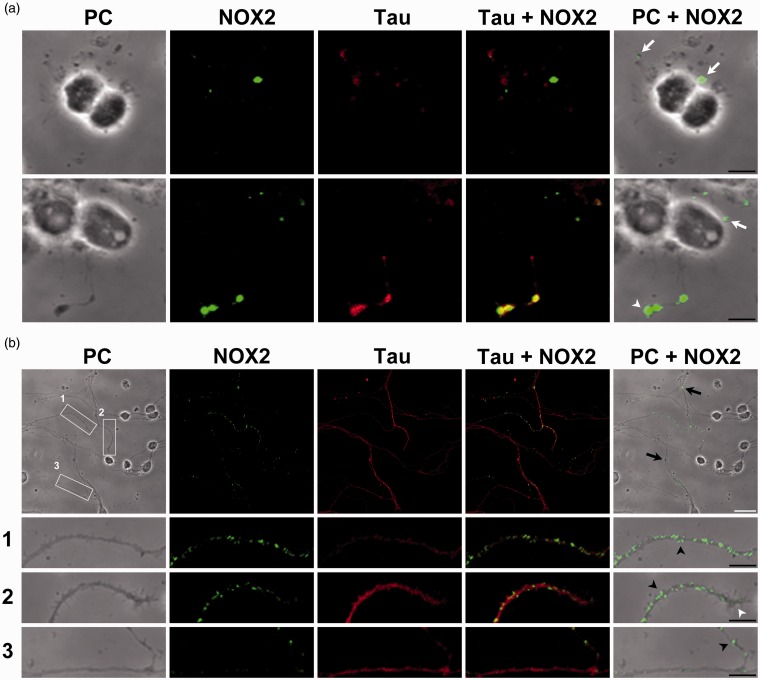
NOX2 is expressed in filopodia and axonal growth cones in developing CGN. Representative confocal micrographs of NOX2 (green) and Tau (red) distribution and phase contrast (PC) micrographs at 0 and 3 DIV. (a) Two representative images of CGN at 0 DIV. White arrows indicate small protrusions and white arrowheads indicate growth cones. (b) A representative micrograph of CGN at 3 DIV. White squares (1 to 3) are shown below as magnified images. CGN were seeded at low density. White arrowheads indicate growth cones, black arrowheads indicate filopodia, and black arrows indicate varicosities (White scale bar, 20 µm; black scale bars, 5 µm). NOX = NADPH-oxidase; CGN = cerebellar granule neurons; DIV = days *in vitro*.

Some studies have highlighted the importance of the compartmentalization of ROS production in cells (Mishina et al., 2011), which may allow ROS to activate particular redox signaling events in specific regions of the cells (Ushio-Fukai, 2009). Accordingly, we studied the localization of NOX2 in developing CGN cultured in depolarizing conditions, based on the finding that this is one of the most abundant homologues in CGN (Figure 4(d) and (e)). Supplementary Figure 2 shows an immunocytochemistry study at normal density, in which the mark of NOX2 is localized in the axons, but no details of the specific location of the label can be appreciated. Therefore, the immunolocalization of NOX2 was carried out in cultures with a lower density than in the rest of the experiments in order to visualize the NOX2-positive structures. This condition did not affect the cell survival (data not shown). Figure 5 shows that NOX2 was mostly distributed in neurites, and it was closely associated with the growth cones at 0 and 3 DIV. Particularly, at 0 DIV, NOX2 was preferentially concentrated in the growth cones of most developing neurites (Figure 5(a)), as well as in some protrusion. In the more advanced stage of development (3 DIV), NOX2 was enriched in filopodia, axonal varicosities, and growth cones (Figure 5(b)); thus suggesting that ROS might be produced in specific regions of the developing neurons. The distribution of Tau, a marker of early neurites, showed a close correlation with NOX2 labeling, particularly at 3 DIV (Figure 5).

Several studies have shown that ROS are produced in axonal growth cones during axonal outgrowth and guidance (Munnamalai and Suter, 2009; Morinaka et al., 2011; Munnamalai et al., 2014). To evaluate ROS compartmentalization, we used an experimental approach that allowed the detection of variations of ROS levels in real time with a high time and spatial resolution. This was achieved by transfecting CGN with the plasmid HyPer that is a genetically encoded fluorescent sensor designed for high specific detection of cytoplasmic H_2_O_2_ (Belousov et al., 2006), which has been suggested to be the most suitable signaling ROS. The ratiometric sensor used has one emission peak at 516 nm and two excitation peaks at 420 nm and 500 nm. The emission fluorescence changes upon exposure to H_2_O_2_; while the excitation peak at 420 nm decreases, the peak at 500 nm increases, which allows us to determine the difference in H_2_O_2_ levels in different regions of the neuron, independently of the amount of protein expressed. We found that H_2_O_2_ is heterogeneously distributed throughout neurons cultured during 1 to 3 DIV under depolarizing conditions (data not shown). Because no apparent differences in the H_2_O_2_ localization were found at 1 to 3 DIV, we used CGN of 2 DIV. At this time, ROS reach their maximum levels, and the plasmid HyPer also reaches its maximum expression.

**Figure 6.**
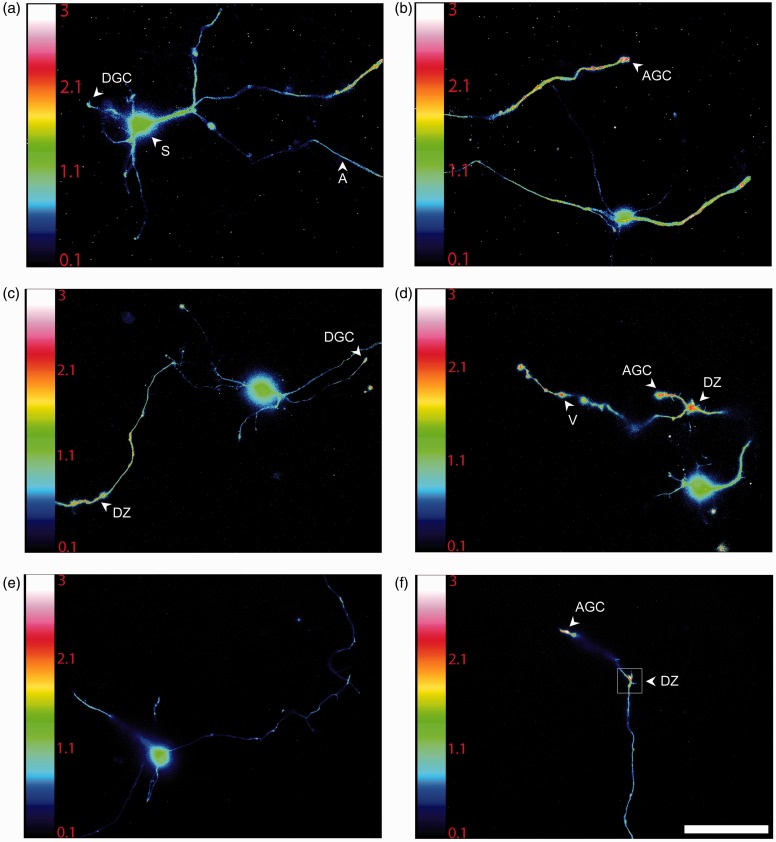

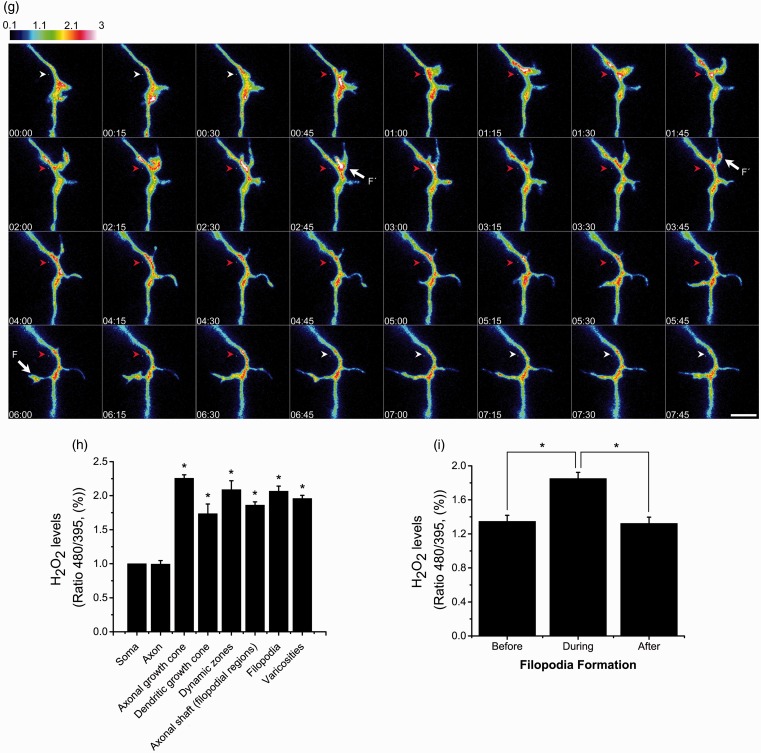
H_2_O_2_ is produced in specific regions of developing CGN. (a to f) Representative micrographs of CGN of 2 DIV transfected with the plasmid HyPer and H_2_O_2_ levels were detected as detailed in Methods. The emission fluorescence was recorded from the excitation wavelengths 480 nm and 395 nm in time-lapse imaging (Supplementary videos). Color scale bars represent the ratio between the excitation wavelengths 480 nm and 395 nm, which represents the regions in the cell where H_2_O_2_ is being produced. Arrowheads indicate: soma (S), axon (A), axonal growth cone (AGC), dendritic growth cone (DGC), dynamic zone (DZ), and varicosities (V) (scale bar, 50 µm). (g) Magnified time-lapse images of the dynamic zone marked in (f). (a) Cell soma of (b). (e) Cell soma of (f). (f) Was captured previously (e) to allow H_2_O_2_ (500 µM) perfusion shown in (f). White arrowheads indicate a region of the axonal shaft previous or posterior to the filopodium formation. Red arrowheads indicate a region of the axonal shaft where a filopodium is present (ASF) and also corresponds to a relative high H_2_O_2_ production area. Arrows indicate filopodia (F´) with relative high H_2_O_2_ production (scale bar, 5 µm). (h) Quantification of H_2_O_2_ levels normalized with respect to the soma (*p < .05, ANOVA nonparametric test, *n* = 42 (A), *n* = 52 (AGC), *n* = 12 (DGC), *n* = 14 (DZ), *n* = 106 (ASF), *n* = 26 (F), *n* = 93 (V)). Data are mean ± *SEM* of 42 neurons registered in time-lapse imaging. (i) Quantification of the fluorescence recorded during filopodia formation normalized with respect to the soma. The fluorescence was measured in ASF during the time before filopodium formation, during the time the filopodium is present and after filopodia retraction. The mean fluorescence in ASF during filopodium formation is significantly different from the mean fluorescence recorded in ASF after and before filopodium formation (*p < .001, Paired *t* test, *n* = 21). H_2_O_2_ = hydrogen peroxide; CGN = cerebellar granule neurons; DIV = days *in vitro*; ANOVA = analysis of variance.

Figure 6(a) to (f) show that, although basal level of H_2_O_2_ was present in all the neuronal structures, there is a clear difference in H_2_O_2_ levels between the soma and the axon and dendrites. H_2_O_2_ is evenly distributed in the soma, and the levels are relatively low (Figure 6(a), (c), and (e)). The levels of H_2_O_2_ in the soma did not change with time under basal conditions, but the fluorescent signal increased when cells were treated with exogenous H_2_O_2_ (Supplementary Figure 3(a) to (d) and Supplementary videos 16–17). In contrast, the distribution of the H_2_O_2_ along the axon shaft was variable under basal conditions; in some areas, the concentration was slightly lower than those observed in the soma, while some other areas showed relatively higher H_2_O_2_ levels than in the soma. In the regions where no filopodia or varicosities are presented, the average level of H_2_O_2_ was approximately equal to the values recorded in the soma (Figure 6(a) to (f)). Interestingly, the axonal zones showing high levels of H_2_O_2_ corresponded to regions with high growing or remodeling activity, such as axonal growth cones and filopodial regions (Figure 6(b) to (d) and (f)). In all cases, these zones showed H_2_O_2_ levels that doubled the levels detected in the soma or other regions of the axon (Figure 6(h)). We also observed that, regardless of the magnitude of movement in the axonal growth cones, these regions always showed high levels of H_2_O_2_ (Supplementary videos 6 to 9). High levels of H_2_O_2_ were also found in dendritic growth cones (Figure 6(a), (c), and (h)). Not all the dynamic regions corresponded to high levels of H_2_O_2_, but also in the varicosities (Figure 6(d) and (h)). The relative levels of H_2_O_2_ in the different regions of the axon are indicated in Table 1.

**Table 1.** Frequency Counts of Structures With Low or High Levels of H_2_O_2_.

Frequency counts of HyPer ratio values (%)							
	0–0.5	0.5–1	1–1.5	1.5–2	2–2.5	2.5–3	3–3.5
Axons	4.761	50	38.095	7.142	0	0	0
AGC	0	0	0	26.923	42.307	28.846	1.923
DGC	0	0	41.666	33.333	25	0	0
DZ	0	0	14.285	35.714	14.285	35.714	0
ASF	0	1.886	19.811	36.792	36.792	3.773	0.943
Filopodia	0	0	3.846	50	34.615	11.538	0
Varicosities	0	0	16.129	33.333	36.559	13.978	0

*Note*. CGN of two DIV were transfected with the plasmid HyPer, and H_2_O_2_ levels were detected as detailed in Methods. The levels of H_2_O_2_ were normalized with respect to the soma. The frequency of different neuronal structures with low or high levels of H_2_O_2_ was counted. Low levels of H_2_O_2_ were considered as those levels that were at least 50% less than the levels of the soma, while high levels of H_2_O_2_ were considered as those levels that exceeded at least 50% the levels of the soma. *n* = 42 (axons), *n* = 52 (axonal growth cone, AGC), *n* = 12 (dendritic growth cone, DGC), *n* = 14 (dynamic zone, DZ), *n* = 106 (axonal shaft in filopodium, ASF), *n* = 26 (filopodia), *n* = 93 (varicosities). Data were acquired from 42 neurons registered in time-lapse imaging.

As mentioned, high levels of H_2_O_2_ are localized in the regions of the axon shaft where filopodia are located (Figure 6(f) to (h)); this was more prominent in regions where filopodia are constantly remodeling (dynamic zones). In these regions, we found clear variations in H_2_O_2_ levels that are restrained to microdomains of the axon shaft where filopodia are growing and retracting. It was generally observed an increase in H_2_O_2_ levels immediately before the filopodia formation, which reached its peak during the process and then dropped to the basal value at the end of the process. In certain cases, high levels were found inside the filopodia, particularly in situations when filopodia were very motile or other protrusions were formed in the filopodia (Figure 6(g) and (i)). These findings are illustrated in Figure 6(g), in which serial images of the dynamic zone are shown.

The observed changes in H_2_O_2_ levels occurred in a time scale of minutes (such as in the case of axonal growth cones, the axon, the soma, and varicosities) or in a range of seconds (such as in the case of dendritic growth cones, dynamic zones, and filopodia). Supplementary Figure 3(e) and (f) show that although H_2_O_2_ levels are continuously fluctuating during the period registered, the levels of H_2_O_2_ are always relatively high. Each image presented in this study has its corresponded supplementary video (see Supplementary videos 1–6) . The scales bars of Figure 6(e) and (f) are different than in Supplementary Figure 3(c) and (f) in order to allow a proper measurement of the ratio during the H_2_O_2_ perfusion.

### Glutathione Maintains Axonal Integrity During the Early Development of CGN

**Figure 7.**
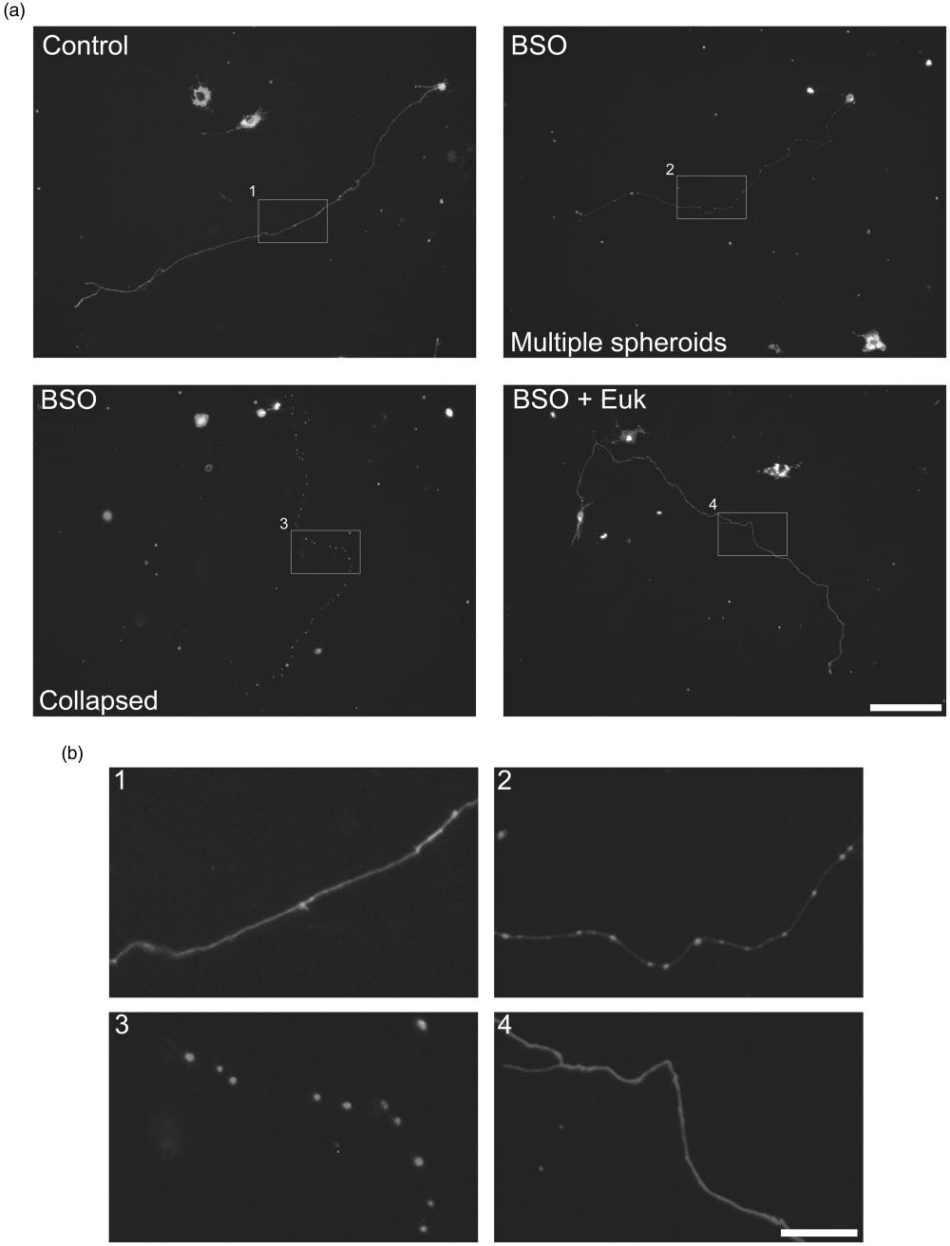

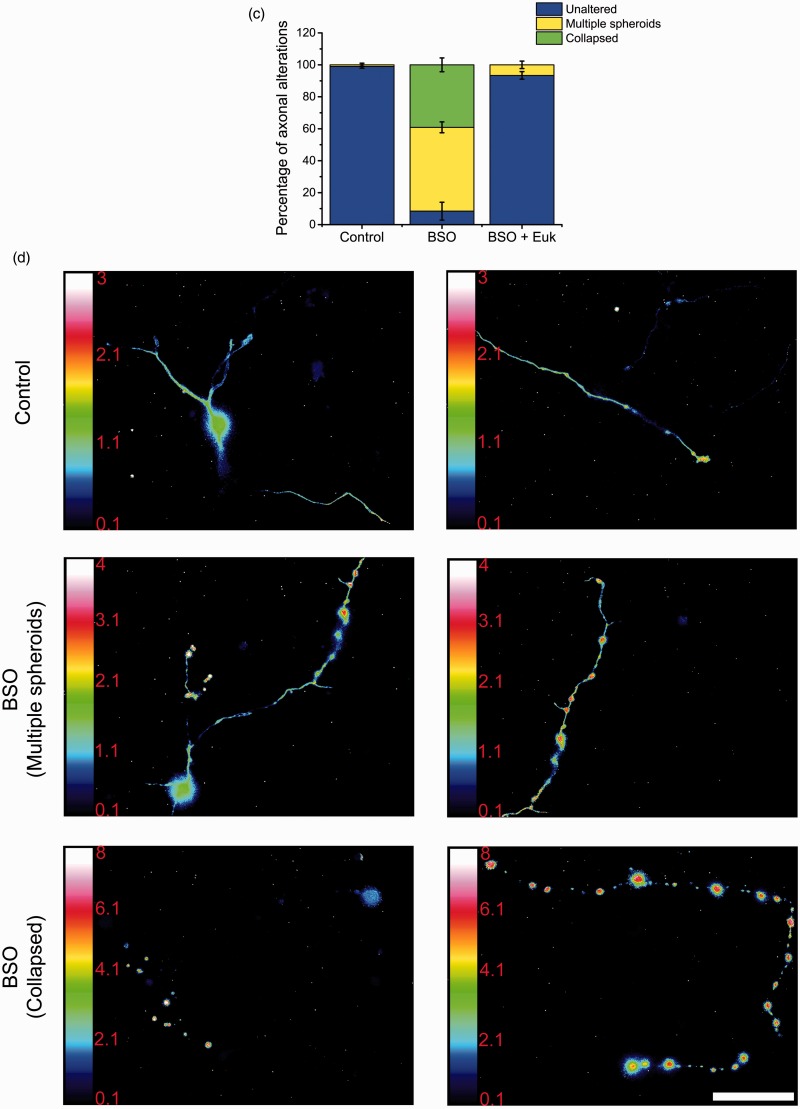
Axonal morphology is altered by glutathione depletion. (a) Representative micrographs of CGN at 2 DIV treated with BSO (100 µM) for 42 hr or with Euk-134 (10 µM) for 18 hr. Cells were labeled with PKH67 (3 µM) before plating and neurites were visualized as detailed in Methods. Two different axonal morphologies were found in CGN treated with BSO, axons containing multiple spheroids, and collapsed axons (scale bar, 100 µm). (b) Magnified images of the indicated areas by white squares in (a) (scale bar, 20 µm). (c) Quantification of axons with morphology altered by BSO treatments. CGN treated with BSO showed a higher percentage of axons with alterations as compared to Control and BSO + Euk-134 (*p* < .05, ANOVA nonparametric test, *n* = 4). (d) Representative micrographs of CGN transfected with the plasmid HyPer as detailed in methods. CGN were treated with BSO (100 µM) for 42 hr and then cells were recorded in time-lapse imaging (scale bar, 50 µm). CGN = cerebellar granule neurons; DIV = days *in vitro*; BSO = buthionine sulphoximine; ANOVA = analysis of variance.

High levels of ROS have been associated with an alteration of cell physiology and structural damage that leads to cell death (Ryter et al., 2007). Also, as we mentioned above, glutathione is a major antioxidant system in neurons. Thus, we evaluated the effect of glutathione depletion by BSO treatment in the structural integrity and H_2_O_2_ levels of CGN axons. To achieve this, we visualized a partial population of CGN stained with PKH67 dye, which allowed us to study individual axons (Supplementary Figure 4). Figure 7(a) shows that 2 DIV CGN treated with BSO during 42 hr show a clear alteration in the axonal structure. Some cells show axons with multiple spheroid-like structures (Figure 7(b)). In other cells, these structures seem to increase in size and number leading to a collapse-like appearance of the axon. No evident alteration in the structure of the somas was detected. The observed effects of BSO are mediated by ROS because the presence of the antioxidant Euk-134 completely prevented the mentioned alterations (Figure 7(a) and (b)). The quantification of the morphological alteration induced by BSO is shown in Figure 7(c). About 40% of the BSO-treated cells showed a collapsed-like axon and 50% corresponded to CGN with little spheroid-like structures. Also, about 10% of the cells with spheroid-like structures were not rescued by Euk-134, and no collapsed-like axons were observed in the presence of the antioxidant, suggesting that the formation of the small spheroids precede the formation of the collapsed-like axons and that neurons show different susceptibility to glutathione depletion. When CGN transfected with the plasmid HyPer were treated with BSO for 42 hr, we found that practically all the spheroid-like structures contained high levels of H_2_O_2_, which was more evident in the collapsed-like axons. No evident increment in H_2_O_2_ levels was detected in the somas (Figure 7(d) and Supplementary videos 10–15). We also observed that axonal continuity was not lost in the collapsed-like axons, as it was possible to observe the continuity of the axons in neurons transfected with HyPer (data not shown).

### NOX2 Regulates Neurite Outgrowth of CGN

**Figure 8.**
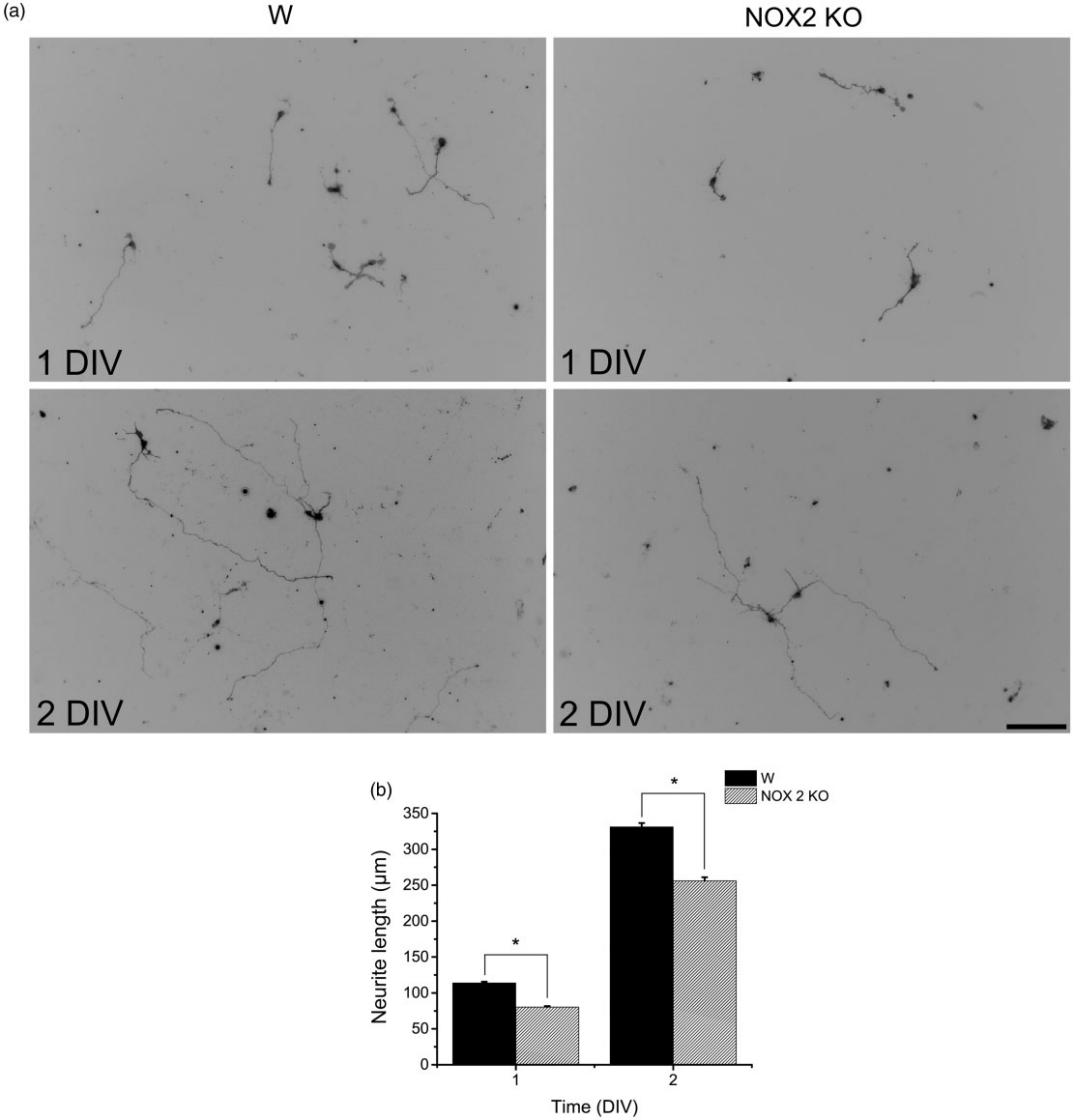

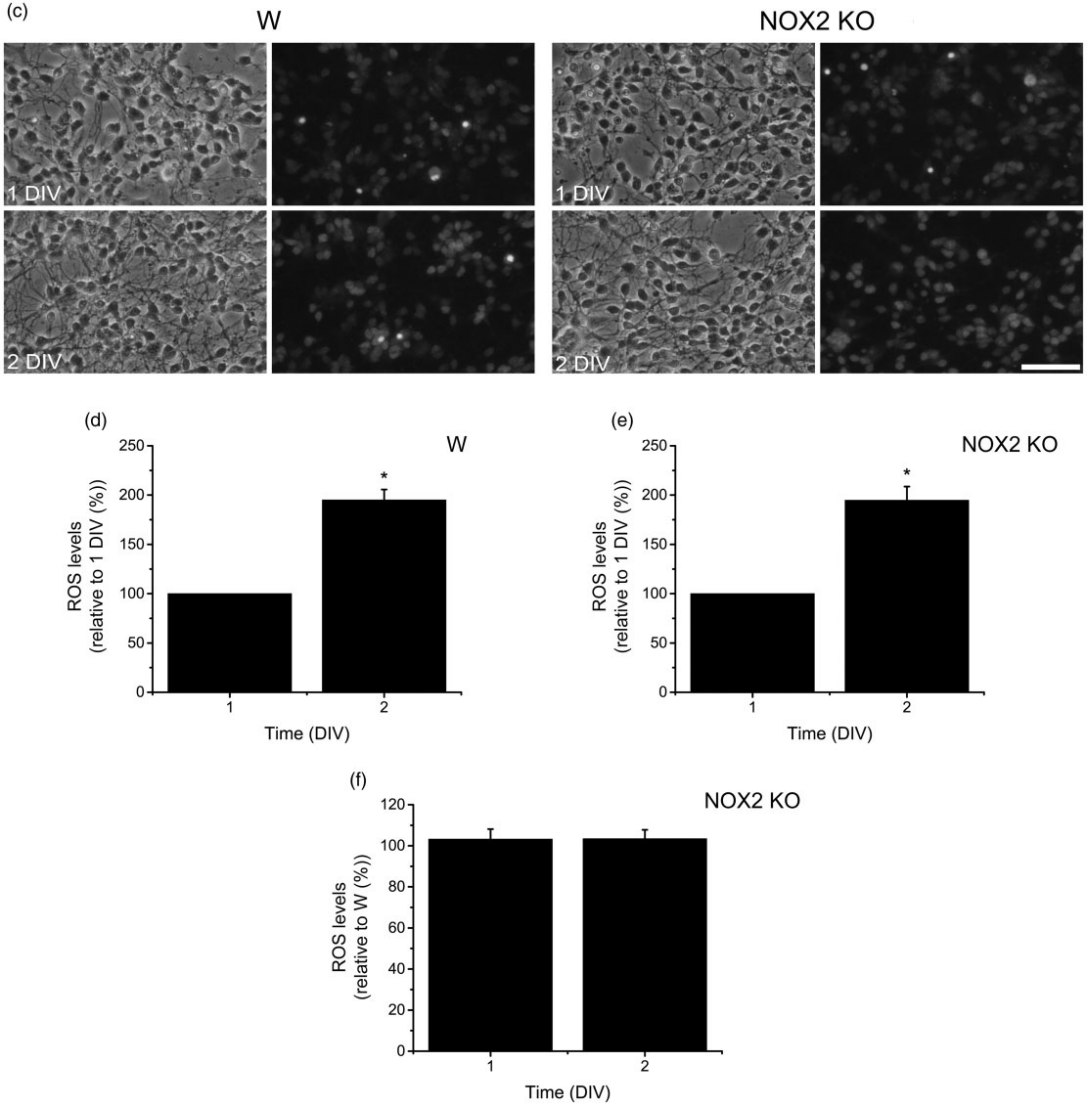
NOX2 regulates axon formation. (a, b) Wild-type (W) and NOX2 KO CGN were labeled with PKH67 (3 µM) before plating and neurites were visualized at 1 and 2 DIV as detailed in Methods. (a) Representative micrographs of CGN labeled with PKH67 at 1 and 2 DIV (scale bar, 100 µm). (b) Quantification of axonal growth of W and NOX2 KO CGN was performed as detailed in Methods. * is significantly different from W at 1 and 2 DIV (*p* < .001, Mann–Whitney U Test, *n* = 2067 and *n* = 1151, respectively). Data are mean ± *SEM*. (c) Representative micrographs of W and NOX2 KO CGN cultured for 1 and 2 DIV and incubated with dihydroethidium as detailed in Methods (scale bar, 100 µm). (d, e) Quantification of ROS levels in W and NOX2 KO CGN cultured from 1 and 2 DIV. * is significantly different from 1 DIV (*p* < .05, Mann-Whitney U Test, *n* = 4). Data were normalized with respect to 1 DIV and are mean ± *SEM*. (f) ROS levels of NOX2 KO CGN at 1 and 2 DIV were compared with respect to W. (No statistical differences were found, Mann–Whitney U Test, *n* = 5). Data were normalized with respect to W and are presented as mean ± *SEM*. NOX = NADPH-oxidase; CGN = cerebellar granule neurons; DIV = days *in vitro*; ROS = reactive oxygen species.

We hypothesized that NOX2 might be involved in the neurite outgrowth of CGN because NOX2 is expressed in growth cones, and NOX inhibitors partially decreased the expression of Tau and MAP2. To address this, we measured the neurite outgrowth and ROS levels in CGN obtained from NOX2 KO and wild-type mice (Figure 8). Under these conditions, we found no apparent differences in wild-type and NOX2 KO CGN in the phase contrast images at 1 and 2 DIV (Figure 8(c)). When CGN were stained with PKH67 and the neurites were measured, we found that CGN neurites from wild-type mouse showed a consistent growth that was markedly increased by about 3 times from 1 to 2 DIV (Figure 8(a) and (b)). In contrast, the neurite outgrowth of NOX2 KO CGN was about 25% (at 1 DIV) and 20% (at 2 DIV) lesser than that observed in CGN from wild-type animals (Figure 8(a) and (b)). When we measured the levels of ROS in wild-type and NOX2 KO CGN, we found that ROS levels increased by twofold from 1 DIV to 2 DIV (Figure 8(c) to (e)). When we compared the levels of ROS between wild-type and NOX2 KO CGN at 1 and 2 DIV, no differences were found (Figure 8(f)).

## Discussion

### ROS and NOX During CGN Development

**Figure 9.**
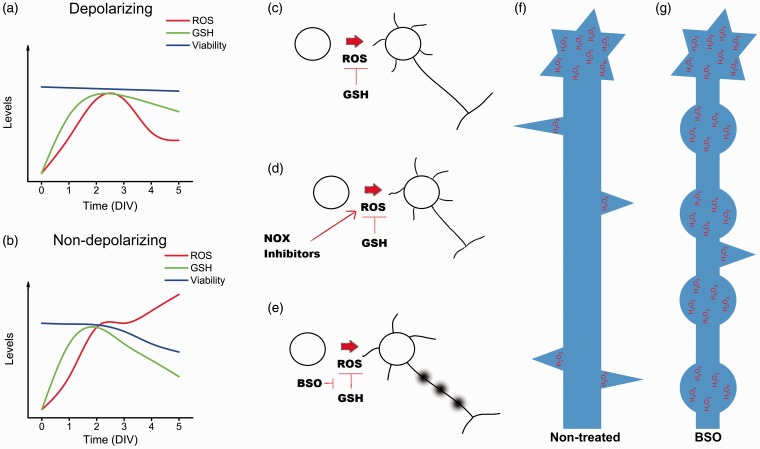
Summary of the principal findings. (a, b) Relationship between the levels of ROS, reduced glutathione and cell survival in CGN under depolarizing and nondepolarizing conditions. During the first 3 DIV, ROS and reduced glutathione increase, reaching the highest levels around 2 DIV. By the third DIV, CGN maintained under depolarizing conditions (a) show a significant decrease in the ROS levels, as well as a moderate reduction of glutathione levels. Cell viability remains unaltered. In CGN cultured in nondepolarizing conditions (b), the levels of ROS remain increasing, while the levels of reduced glutathione decrease and the cell survival is compromised. (c to e) Effect of ROS in the development of CGN. (c) During normal development, the levels of ROS are regulated by reduced glutathione. Also, (d) when ROS production is decreased by NOX2 inhibition, the development of CGN is altered, as indicated by a low expression of the neuronal markers, Tau and MAP2, as well as a reduced axonal growth. (e) In contrast, the reduction of the levels of glutathione leads to an alteration in the axonal development and cell death. (f) The H_2_O_2_ produced in CGN is mainly localized in the axons; this H_2_O_2_ is associated with the formation of filopodia and with axonal growth cone dynamics. (g) Glutathione depletion leads to the formation of multiple spheroid-like structures in the axon that are rich in H_2_O_2_. ROS = reactive oxygen species; CGN = cerebellar granule neurons; DIV = days *in vitro*; NOX = NADPH-oxidase; H_2_O_2_ = hydrogen peroxide.

In this study, we found that ROS production is regulated throughout CGN development. In this regard, we propose that CGN development could be divided into two distinct phases. In the first phase, ROS increase during the first 2 DIV and remain high until 3 DIV, which is independent of the state of depolarization. In the second phase, the levels of ROS are dependent on the state of depolarization; in depolarizing conditions, ROS levels diminish at 4 DIV and then remain low during the subsequent days, while in nondepolarizing conditions, ROS levels continue increasing at 4 and 5 DIV (Figure 9(a) and (b)). During these two phases, CGN undergo different developmental processes. During the first phase, CGN initiate the biochemical (Gallo et al., 1987) and morphological (Powell et al., 1997) processes of maturation, and at 3 DIV, most CGN have already developed the axon and multiple short dendrites (de la Torre-Ubieta et al., 2010). The second phase is coincident with a dependence of CGN survival on depolarization (Gallo et al., 1987) and dendrite maturation (Shalizi et al., 2006; Ramos et al., 2007).

Our results show that, in spite of the high levels of ROS observed at 2 and 3 DIV, CGN viability is not compromised regardless the state of depolarization. In contrast, during the second phase, our data show a correlation between low ROS levels and CGN survival in depolarizing conditions, but in nondepolarizing conditions, high ROS levels are associated with a reduction of the metabolic activity and cell survival (Figure 9(a) and (b)). In this regard, we have previously shown that ROS could be signals that trigger the process of apoptotic cell death of CGN at 8 DIV when CGN are switched from a depolarizing condition to a nondepolarizing condition (Ramiro-Cortes and Moran, 2009; Ramiro-Cortes et al., 2011). Together, these results suggest that the regulation of ROS during CGN development may be critical for the survival of CGN during the second phase.

### Glutathione Regulation During CGN Development

Here, we also addressed the possibility that glutathione could be responsible for regulating the levels of ROS during development, this is supported by the idea that the balance between ROS production and the expression and activity of the antioxidant systems determine ROS levels in the cells. It is known that during nervous system development, neurons and glial cells contain glutathione, and by the postnatal Day 5, most neurons have decreased the levels of glutathione, while olfactory mitral and granule cells, CGN, and dorsal root ganglion neurons consistently retain high levels of glutathione throughout development and in adulthood (Beiswanger et al., 1995). Moreover, glutathione deficient mice die before the embryonic Day 8.5 by massive apoptosis (Shi et al., 2000), and mice lacking of glutathione peroxidase 4 (GPx4) die before the embryonic Day 8 also by an increase in apoptosis (Yant et al., 2003). Interestingly, while GPx4 is expressed in neurons, glial cells are devoid of GPx4 (Savaskan et al., 2007); furthermore, it seems that the developing brain constitutes a major site for GPx4 expression, while the specific suppression of GPx4 between the embryonic Days 7.5 and 10.5 led to microcephaly and abnormal hindbrain development (Borchert et al., 2006). These studies suggest a fundamental role of glutathione during nervous system development.

In this study, we found changes in the levels of glutathione content during CGN development. In the first phase, when ROS levels are relatively high, the glutathione content increases independently of the state of depolarization; in general, during the time when ROS are in their highest levels, the glutathione content are also in its highest levels. One possibility to explain this observation could be associated with a mechanism to balance the levels of ROS required for optimal redox signaling and to prevent the deleterious effects of ROS during the first phase. The major increase of glutathione content occurred from 0 DIV to 1 DIV which precedes the major increase observed in ROS (Figure 9(a) and (b)). Thereby, these results might suggest that glutathione content in CGN development responds to an internal genetic program of the cells, which is independent of the trophic conditions and ROS levels. The idea is supported by the fact that *in vivo*, the glutathione content in the developing cerebellum increases by the time when CGN precursors begin their process of differentiation CGN (Nanda et al., 1996; Rice and Russo-Menna, 1998; Komuro and Yacubova, 2003).

It is known that chronic treatments with BSO and oxygen in rat-pups lead to a high rate of apoptosis in the hippocampus, cerebellum, basal forebrain, and striatum (Taglialatela et al., 1998). In this regard, our results demonstrate that the increase in the glutathione content is necessary to allow CGN development because glutathione depletion at 2 DIV drastically reduces CGN survival, but it does not affect CGN survival in later stages of CGN development. This process could be explained by two major mechanisms of action of glutathione, one is the interaction of glutathione with proteins, which modifies signaling pathways during variations in ROS homeostasis (Grek et al., 2013) and by a second mechanism that involves the antioxidant actions of glutathione. The fact that the antioxidant Euk-134 completely rescues CGN viability under glutathione depletion strongly suggests that the effect of glutathione in CGN survival is due to its antioxidant properties.

During the second phase, the levels of glutathione content are dependent on the state of depolarization. In depolarizing conditions, glutathione remains high from 3 to 5 DIV, a time when ROS levels decrease markedly. In contrast, in nondepolarizing conditions, the glutathione content diminishes since 4 DIV, which correlates with the observed decrease in CGN survival and high ROS levels (Figure 9(a) and (b)). This decrease in glutathione content can be considered an early hallmark of the cell death progression (Franco and Cidlowski, 2012). To rule out the possibility that the decrease of glutathione content is responsible for the cell death observed during the second phase in nondepolarizing conditions, we diminished the levels of glutathione with BSO; however, although glutathione content was importantly reduced with BSO treatment, the cell viability at the incubation tested times did not alter CGN survival at 5 DIV under depolarizing conditions, indicating that a decrease in glutathione content is not sufficient to trigger cell death in developing CGN and that the decrease in glutathione content during the second phase in nondepolarizing conditions is probably a consequence of the cell death process.

### ROS and NOX in CGN Maturation

The possibility that ROS are required for CGN development in the first phase is supported by our results showing that antioxidant conditions markedly diminished the levels of Tau and MAP2 proteins. Interestingly, we found that the antioxidants Ebselen and Euk-134 only reduced MAP2 expression at 2 DIV, while the NOX inhibitors, AEBSF, and apocynin diminished the levels of Tau at 1 and 2 DIV, suggesting a more specific action of the ROS produced by NOX activity in this process (Figure 9(c) and (d)). None of these treatments diminished the expression of these proteins at 3 DIV, which could be explained by the fact that between 2 and 3 DIV the levels of these two proteins are not significantly increased under control conditions or that by this time the maturational process of CGN is no longer dependent on ROS.

It is noteworthy to mention that, based on the proposed mechanism of inhibition of AEBSF and apocynin, their primary target would be the NOX1 and NOX2 homologues (Jaquet et al., 2009). However, because the effect of these inhibitors is only partial, in this study, we cannot exclude the contribution of other NOX homologues and other sources of ROS in the CGN development. Also, although we cannot discard that part of the effects of apocynin or AEBSF could be due to unspecific actions, their effects are validated with the use of a second NOX inhibitor (apocynin or AEBSF). Thus, we think that the results obtained using separately both inhibitors with similar results may indicate that NOX is involved in the production of ROS required for CGN maturation.

Our results are in accordance with previous observations indicating that ROS produced by NOX enzymes may act as signaling molecules in the regulation of the neuronal differentiation of PC12 cells. In these cells, it is well known that the neurotrophin NGF induces the expression of different neuronal markers such as βIII-tubulin, GAP-43, and neurofilament L through the activation of the receptor TrkA which leads to the activation of the signaling pathway Ras/Raf/MEK/ERKs (Kaplan and Miller, 2000; Patapoutian and Reichardt, 2001). Besides, NGF induces an increase in the levels of ROS produced by NOX after 10 min of treatment (Suzukawa et al., 2000). These ROS induce the phosphorylation of TrkA through the inhibition of protein tyrosine phosphatases and the formation of complexes with the scaffold proteins Shc, Grb2, and Sos, which are required for the activation of the MAPK pathway (Kamata et al., 2005). On the other hand, NGF also induces the phosphorylation of ERKs after 30 min, which induces the expression and activity of the mitochondrial super oxide dismutase (MnSOD), and the H_2_O_2_ resulting from the MnSOD activity induces a second phosphorylation of ERKs, which is essential for PC12 differentiation (Cassano et al., 2010).

Further studies have shown that NOX enzymes mediate the neuronal differentiation in different models (Wang et al., 2007; Kennedy et al., 2010; Nitti et al., 2010; Le Belle et al., 2011). In the present study, we found that NOX shows a pattern of activity similar to the ROS levels observed during the first and second phases of CGN development in depolarizing conditions. Different NOX homologues could contribute to the observed NOX activity, which is in line with our findings about the expression of NOX1 and NOX2 enzymes that are highly regulated throughout CGN development. Overall, in the first phase, the mRNA levels of both NOX1 and NOX2 are upregulated, while in the second phase, they are downregulated. Interestingly, each NOX homologue showed a slightly different pattern of expression, which suggests that these enzymes might have distinct roles during CGN development.

It is important to note that the expression of NOX2 follows more closely both the total NOX activity and the ROS levels. Furthermore, the mRNA levels of NOX2 are considerably more abundant than mRNA NOX1 levels. Thereby, we hypothesize that NOX2 could be the main source of the ROS produced during CGN development. However, we found no differences in the levels of ROS measured by dihydroethidium between NOX2 KO and wild-type CGN at 1 DIV and 2 DIV. Thus, these results suggest that NOX2 is not responsible for the increase of ROS observed during the first phase of CGN development. Nevertheless, we cannot discard a possible compensatory mechanism in the NOX2 KO CGN, which has been demonstrated in other preparations where an upregulation of NOX4 occurs in NOX2 KO mice in cells where NOX2 gene has been silenced (Pendyala et al., 2009) or when an indirect downregulation of NOX2 leads to an overexpression of NOX4 (Sedeek et al., 2010).

Despite the fact that ROS levels measured by dihydroethidium were not affected in NOX2 KO CGN, we found a slight decrease in the length of CGN axons at 1 DIV and 2 DIV, which corroborates the importance of NOX2 during CGN development. Our findings that NOX2 KO CGN showed a reduction of neurite outgrow is in agreement with other studies carried out in *Aplysia* neurons that shows that NOX inhibitors reduce actin flow and its assembly at the leading edge of the axonal growth cone, leading to a decrease of the neurite outgrowth (Munnamalai and Suter, 2009). The regulation of NOX2 in the axonal growth cones of *Aplysia* neurons seems to be bidirectional because the stimulation of NOX2 produces H_2_O_2_, which regulates F-actin dynamics and neurite outgrowth; on the other hand, the stimulation of neurite outgrowth induces the proximity of the cytoplasmic subunit of NOX2, p40phox, to its catalytic subunit, gp91phox, indicating that the regulation of cytoskeleton dynamics affects NOX2 activity (Munnamalai et al., 2014).

The relevance of NOX2 in the nervous system development is strengthened by the alterations of the brain physiology observed in NOX2 KO mice. These animals display deficits in hippocampal synaptic plasticity and mild impairments in cognitive function, as well a slight deficiency in motor learning (Kishida et al., 2006), which might be attributed to an altered function in either the cerebellar cortex or the deep cerebellar nuclei (Caston et al., 1995). Moreover, these mice have less proliferating cells in the subventricular zone, less migrating cells and fewer neuronal differentiation in the olfactory bulb than in wild-type mice (Le Belle et al., 2011). However, it has not been reported if the alterations in motor behavior observed in NOX2 KO mice are due to aberrations in the formation of cerebellar circuits or alterations in the synaptic plasticity and function. In a previous study, we found that the treatment of developing rats with apocynin or with the antioxidant MnTmPyP, produced alterations in the cerebellar foliation as well as a deficiency in motor behavior, measured as alteration in the rod walking and Rotarod tests (Coyoy et al., 2013). The results obtained in the present study suggest that part of the actions of ROS and NOX could be related to neurite development during cerebellar cortex formation.

### H_2_O_2_ and NOX Localization

In line with the above findings, we explored the possibility that H_2_O_2_ were produced locally in developing CGN, as well as the possible contribution of glutathione as a regulator of H_2_O_2_. Although it is not completely understood how redox signaling occurs, there is increasing evidence indicating that the physiological function of ROS, and particularly H_2_O_2_, might occur by the activation of specific signaling pathways through reversible redox modifications of proteins in subcellular compartments (Chen et al., 2009; Ushio-Fukai, 2009; Gough and Cotter, 2011; Mishina et al., 2011; Kaludercic et al., 2014). Interestingly, we found different regions of the developing axons and dendrites where H_2_O_2_ is continuously produced. These regions apparently correspond to those regions where NOX2 is expressed. For example, NOX2 is enriched in filopodia, either in the axonal shaft where filopodia are located or inside the filopodia, coincident with the microdomains where H_2_O_2_ is continuously produced (Figure 9(f)), which suggest that both NOX2 and H_2_O_2_ might be related to filopodial dynamics. Moreover, we also observed that the increase of H_2_O_2_ inside the filopodia occurs previous to the filopodia elongation and that after the process of filopodia retraction, the amount of H_2_O_2_ returns to basal levels. Together, these results strongly suggest that H_2_O_2_ is involved in filopodia formation and that NOX2 might be a possible source for the H_2_O_2_ produced. This idea is supported by previous studies showing that ROS control actin dynamics (Munnamalai and Suter, 2009; Sakai et al., 2012; Lee et al., 2013; Munnamalai et al., 2014). In neutrophils, the physiological levels of H_2_O_2_ produced by NOX2 negatively regulate the actin polymerization by inducing actin glutathionylation, while the overexpression of glutaredoxin 1, which is the enzyme that catalyzes actin deglutathionylation, leads to multiple pseudopodia formation (Sakai et al., 2012). In macrophages, the formation of filopodia and actin polymerization are regulated by the reversible oxidation of two methionine residues by MICAL1 and MICAL2; the oxidation of these residues leads to actin disassembly, while the reduction of these residues by the methionine-R-sulfoxide reductase B1 lead to actin assembly, which in conjunction orchestrate actin dynamics and macrophage function (Lee et al., 2013). Therefore, we speculate that redox reactions might control filopodia formation in developing CGN.

On the other hand, the localization of NOX2 in the axonal growth cones also correlates with the observed high levels of H_2_O_2_. This result suggests that H_2_O_2_ might be related to axonal growth cone dynamics and that H_2_O_2_ could be produced by NOX2, which is in line with the observation that NOX2 KO CGN showed less axonal growth than the wild-type CGN (Figure 9(d) and (f)). This association has been previously examined in *Aplysia* neurons, where the H_2_O_2_ produced by NOX2 modulates f-actin dynamics and promotes axonal growth (Munnamalai et al., 2014). On the other hand, it has also been shown that H_2_O_2_ negatively regulates axonal growth. In dorsal root ganglion neurons, semaphorin3A induces growth cone collapse by the regulation of cytoskeleton through CRMP2. Semaphorine3A induces the generation of H_2_O_2_ in the axonal growth cone through MICAL, which oxidizes CRMP2 and induces the formation of a transient disulfide-linked homodimer between the cysteines 504 of two CRMP2 proteins. Then, this homodimer is reduced by thioredoxin that forms a disulfide bond with one molecule of CRMP2. This complex is crucial for CRMP2 phosphorylation by GSK3-β, which ultimately produces the growth cone collapse of these neurons (Morinaka et al., 2011). Although it is not completely understood the regulation of the axonal growth cone dynamics by H_2_O_2_, it seems that multiple ROS sources are localized in this region, and it is clear that ROS regulate the mechanisms responsible for the morphological organization of these structures; thereby, further studies are required to asses these issues.

Finally, we explored the possibility that axonal integrity were altered in the absence of glutathione. Interestingly, while we found no evident alterations in the neuronal soma, we observed a severe aberrant axonal alteration in CGN treated with BSO that conduces to axonal degeneration followed by cell death, which suggest that ROS levels are critical for both axonal formation and integrity (Figure 9(e) and (g)). The spheroid-like structures formed in BSO treatments are possibly formed by the dysregulation of the H_2_O_2_ production in the axon because these structures are enriched in H_2_O_2_ and their formation is drastically inhibited by the antioxidant treatment. The spheroid-like structures found in CGN treated with BSO resemble the structures found in a classic model of axonal degeneration. This could have clinical implications because axons degenerate before cell bodies in different neurodegenerative diseases such as amyotrophic lateral sclerosis, Alzheimer's disease, Parkinson's disease, and Huntington's disease, among others (Coleman, 2005; Conforti et al., 2014), which has been related to a process of oxidative stress (Barnham et al., 2004). Thereby, we speculate that the dysregulation of the mechanisms that regulate ROS during neuronal development could be similar to those that are involved in neurodegenerative diseases.

## Conclusions

In the present study, we showed evidence suggesting that ROS regulate different aspects of CGN development. During the first 3 DIV, ROS increased which is necessary for CGN maturation. If the production of these molecules is not regulated in the subsequent days due to the trophic conditions (i.e., low potassium), ROS lead to CGN death. Before the peak of ROS, glutathione content increased, which seems to be required for CGN to complete their development because the pharmacologically induced depletion of glutathione at this time conduced to CGN death. Most of the ROS produced at this stage are probably produced by NOX2, which is mainly localized in filopodia and growth cones. These regions correspond to microdomains in which H_2_O_2_ is continuously produced. In line with these results, neurite outgrowth is less in NOX2 KO CGN than in wild-type CGN, which suggest that NOX2 is important during the early stages of CGN development. Finally, the pharmacological depletion of glutathione demonstrated the importance of glutathione as a regulator of the H_2_O_2_ produced in CGN axons and the importance of ROS during the axonal morphogenesis. Together these results suggest that the H_2_O_2_ produced by NOX2, in combination with glutathione, controls axonal morphogenesis in CGN, which contribute to our understanding about neuronal development.

## Summary

The antioxidant systems involving glutathione and the pro-oxidant NOX2 system regulates the reactive oxygen species levels involved in the axonal morphogenesis and cell survival of cerebellar granule neurons during the early stages of development in an *in vitro* model.

## Author Note

This study is part of the requirements for the PhD degree in Biomedical Sciences of Mauricio Olguín-Albuerne at Universidad Nacional Autónoma de México.
